# Insulin signaling represents a gating mechanism between different memory phases in *Drosophila* larvae

**DOI:** 10.1371/journal.pgen.1009064

**Published:** 2020-10-26

**Authors:** Melanie Eschment, Hanna R. Franz, Nazlı Güllü, Luis G. Hölscher, Ko-Eun Huh, Annekathrin Widmann

**Affiliations:** 1 Department of Biology, University of Konstanz, Konstanz, Germany; 2 Department of Molecular Neurobiology of Behavior, University of Göttingen, Göttingen, Germany; Katholieke Universiteit Leuven, BELGIUM

## Abstract

The ability to learn new skills and to store them as memory entities is one of the most impressive features of higher evolved organisms. However, not all memories are created equal; some are short-lived forms, and some are longer lasting. Formation of the latter is energetically costly and by the reason of restricted availability of food or fluctuations in energy expanses, efficient metabolic homeostasis modulating different needs like survival, growth, reproduction, or investment in longer lasting memories is crucial. Whilst equipped with cellular and molecular pre-requisites for formation of a protein synthesis dependent long-term memory (LTM), its existence in the larval stage of *Drosophila* remains elusive. Considering it from the viewpoint that larval brain structures are completely rebuilt during metamorphosis, and that this process depends completely on accumulated energy stores formed during the larval stage, investing in LTM represents an unnecessary expenditure. However, as an alternative, *Drosophila* larvae are equipped with the capacity to form a protein synthesis independent so-called larval anaesthesia resistant memory (lARM), which is consolidated in terms of being insensitive to cold-shock treatments. Motivated by the fact that LTM formation causes an increase in energy uptake in *Drosophila* adults, we tested the idea of whether an energy surplus can induce the formation of LTM in the larval stage. Suprisingly, increasing the metabolic state by feeding *Drosophila* larvae the disaccharide sucrose directly before aversive olfactory conditioning led to the formation of a protein synthesis dependent longer lasting memory. Moreover, formation of this memory component is accompanied by the suppression of lARM. We ascertained that insulin receptors (InRs) expressed in the mushroom body Kenyon cells suppresses the formation of lARM and induces the formation of a protein synthesis dependent longer lasting memory in *Drosophila* larvae. Given the numerical simplicity of the larval nervous system this work offers a unique prospect to study the impact of insulin signaling on the formation of protein synthesis dependent memories on a molecular level.

## Author summary

Memories enable organisms to deal with novelties and unpredictable complexities in an ever-changing environment by allowing past experiences to influence future behaviors. However, every memory has an energetic cost; some more, some less. In *Drosophila*, two long-lasting and mutually exclusive memory phases can be distinguished: anaesthesia-resistant memory (ARM) and long-term memory (LTM). The protein synthesis dependent formation of LTM is accompanied by a drastic increase in energy uptake and is disabled after reduced food availability. To understand the molecular underpinnings of how the brain disables LTM, we used a reductionistic approach. *Drosophila* larvae are equipped with the cellular and molecular pre-requisites of forming different memory component. However, the existence of LTM in the larval stage of *Drosophila* remains elusive. Theoretically, the emerging costs of a protein synthesis dependent LTM in larvae exceed the potential benefit since learned information becomes obsolete through metamorphosis. With this in mind, we tested whether an energy surplus can induce the formation of a protein synthesis dependent longer lasting memory in *Drosophila* larvae, and correlate this with molecular events in identifiable neurons. Indeed, an energy surplus prior to aversive Pavlovian conditioning successfully leads to the formation of a protein synthesis dependent memory and to the inhibition of ARM. We further demonstrate that this state-dependent switch between different types of memories is reliant on the activity of the *rutabaga*-encoded adenylate cyclase and insulin receptors expressed in the mushroom body Kenyon cells.

## Introduction

Harboring the ability to deal with novelties and unpredictable complexities provides the key to successfully adapt to unforeseen events in an ever-changing environment. Therefore, one of the most outstanding capabilities of higher evolved organisms is the capacity to constantly learn new tasks, integrate new skills and preserve them as memory entities. However, establishing a memory is a highly complex and dynamic process. Apart from the involvement of multilayered neuronal circuitries and cellular machineries [1], the capacity to form memories comes with energetic costs since activation and maintenance of synaptic connections involved in integrating, storing and retrieving information are energy demanding [2,3]. These circumstances can either lead to trade-offs with other phenotypic traits or to learning and memory impairments, when available energy resources are restricted [4,5]. For example, trade-offs between learning abilities and longevity and competitive abilities in *Drosophila* [6–8], reduced foraging skills in bumble bees [9], delayed juvenile development in mites [10], and decreased fecundity in guppies [11] and butterflies [12] have been described. Moreover, honeybees experience significant costs for learning and show a memory deficit being energetically stressed [13]. On the other hand, formation of LTM led to reduced resistance to food and water stress in *Drosophila* [14] and during food deprivation the formation of energetically costly LTM is disabled [15].

A general feature of memory formation across species is the parallel and chronologically ordered occurrence of distinct short-, intermediate-, and/or long-lasting memory phases [1]. In adult *Drosophila*, four temporally distinct memory phases have been characterized, in which one phase can be further subdivided [16,17]. Thereby, LTM and ARM represent longer lasting memories that are resistant to anesthetic disruption but are mutually exclusive and distinguished by their dependence on *de novo* protein synthesis; LTM requires protein synthesis whereas ARM does not [18,19]. In adult *Drosophila* the formation of LTM, by protein synthesis dependency [18], causes an increase in energy uptake [20]. Under conditions of reduced food availability, the brain disables the formation of costly LTM and favors the formation of ARM [15]. One hypothesis proposes that “neuronal gating mechanisms” prevent adult *Drosophila* from forming energetically costly LTM under critical nutritional circumstances [20,21].

The larval stage of *Drosophila* has emerged as a favorable model system for studying learning and memory [22] because of the relative simplicity of the brain, for which the complete synaptic connectome is known [23,24]. Olfactory memory during the larval stage of *Drosophila* also consists of different memory phases [24]. For example, after classical aversive Pavlovian conditioning, during which larvae associate an odor with an aversive high salt stimulus [25,26], at least two co-existing memory phases have been distinguished: a labile larval short-term memory (lSTM) and lARM that are encoded by separate molecular pathways [25]. Although, *Drosophila* larvae possess cellular and molecular pre-requisites of potentially forming a protein synthesis dependent long-term memory (LTM) [24], the existence of a protein synthesis dependent LTM remains still elusive. Memorizing behavioral adjustments based on previous experience depends on the balancing of costs and benefits: only relevant information should be stored into energetically costly, protein synthesis dependent longer lasting memories, whereas less reliable information should be disregarded. Accordingly, the formation of protein synthesis dependent longer lasting memories in larvae would represent an unnecessary expenditure, since larval brain structures are completely rebuilt during metamorphosis–meaning any plastic changes that occur due to learning might be lost in the re-wiring of the brain. However, we were able to show that by elevating the energetic state of larvae by feeding sucrose before conditioning, larvae are able to successfully form aversive *de-novo* protein synthesis dependent (PSD) memory, which is longer lasting. Conversely, we show that such a protocol inhibits the formation of lARM. We were additionally able to demonstrate that the process of PSD memory formation depends on the activity of the *rutabaga* (*rut*) adenylate cyclase (AC), and that insulin receptors (IRs) in the mushroom body Kenyon cells (MB KCs) gate the state-dependent switch between lARM and a PSD memory.

## Results

### Sucrose consumption specifically suppresses lARM

**Fig 1:**
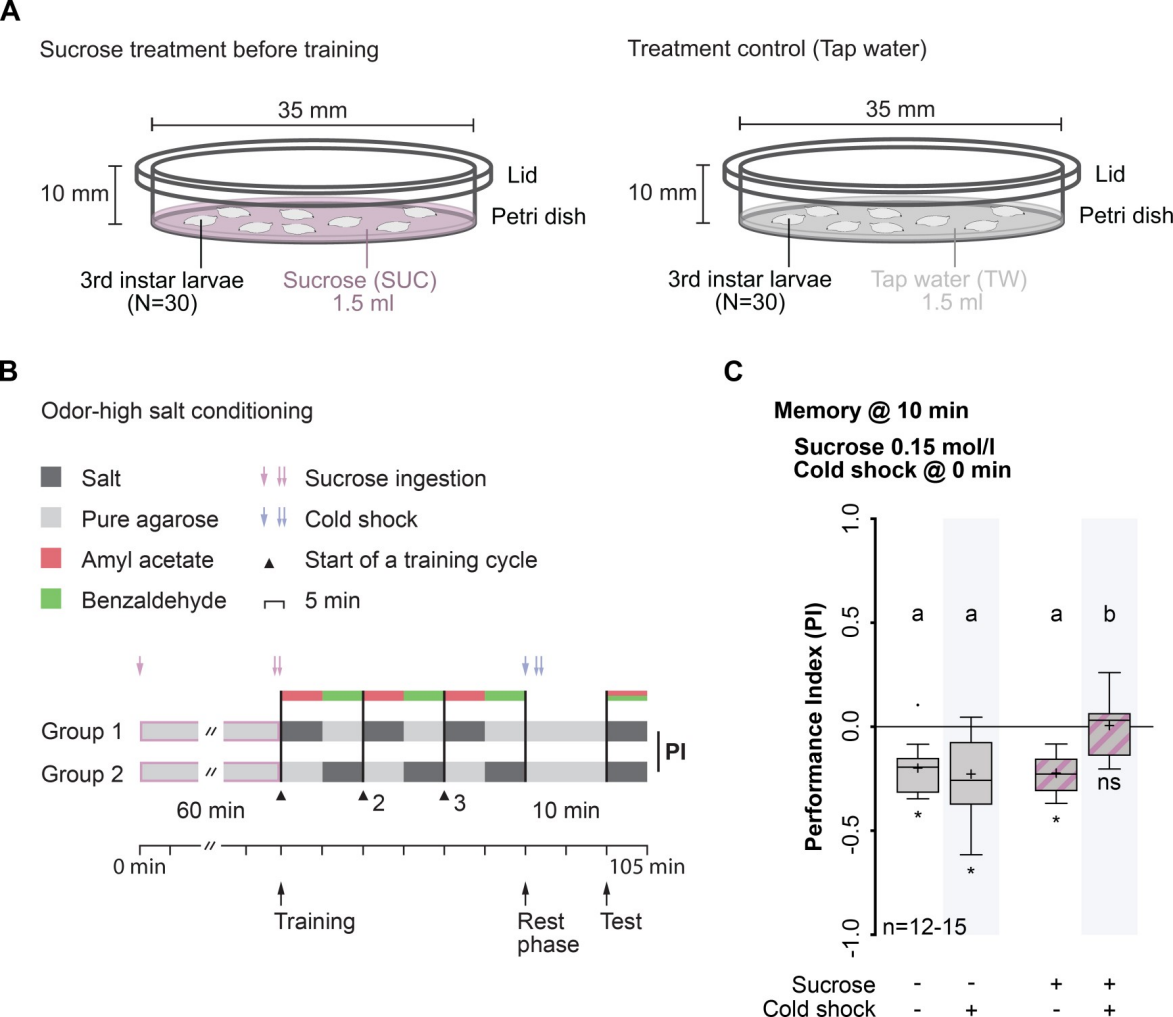
Sucrose consumption specifically suppresses lARM. (A) Schematic illustration of the manipulation of the metabolic state via sucrose feeding. Larvae were placed in a Petri dish filled with agarose containing either sucrose solution (SUC) (left) or tap water (TW) (right) for 60 min. (B) Schematic illustration of experimental principles and odor-high salt conditioning using a two-odor reciprocal training paradigm. After feeding on 0.15 M SUC for 60 min (1 red arrow, start of feeding; 2 red arrows, end of feeding) two groups of 30 larvae were trained reciprocally with 3 training cycles without temporal gaps. Group 1 received the first odor n-amyl acetate (AM) paired with an aversive reinforcer (high salt concentration) while the second odor benzaldehyde (BA) was presented alone (AM+/BA). Group 2 received the reverse contingency (AM/BA+). Subsequently, larvae received a cold shock treatment for 1 min (1 blue arrow, start of cold shock treatment; 2 blue arrows, end of cold shock treatment). Memory was tested 10 min later by calculating a Performance Index (PI). (C) After sucrose consumption, wild-type larvae showed a complete memory loss upon cold shock treatment. Larvae that consumed sucrose but did not receive a cold shock treatment showed aversive olfactory memory, comparable to larvae that did not consume sucrose independently of cold shock treatment. Memory performance above the level of chance was tested using Bonferroni-corrected one-sample t-tests (ns p≥0.05/4; * p<0.05/4; adjusted significance level α). Differences between the groups were determined using two-way ANOVA followed by Bonferroni *post-hoc* pairwise comparisons. Lowercase letters indicate differences between groups (p<0.05). For more statistical details see also S1 Table and S3 Table. Data are shown as Tukey box plots; line, median; cross, mean; box, 75th-25th percentiles; whiskers, 1.5 interquartile range; small circles, outlier (n≥8).

We first asked whether an increase in nutritional energy through carbohydrate uptake over a short period of time affects lARM. To tackle this question, we tested the memory performance of third instar, wild-type larvae trained using a previously described three-cycle aversive olfactory conditioning protocol [25], which was here additionally preceded by sucrose feeding for 60 min—to elevate the energetic state—and followed by an anesthetizing cold shock treatment (4°C) for 1 min [25]—to isolate lARM **(**Fig 1A and 1B). The memory tested 40 min after training onset (10 min after training offset) in larvae that consumed sucrose was indistinguishable from that of control larvae that consumed only tap water (Fig 1C, S3 Table). This memory was completely abolished after cold shock treatment (Fig 1C, S1 Table). Therefore, we concluded that lARM is not detectable after sucrose consumption anymore. It is unlikely that this memory phase is a residual lSTM, because it is well-established that lSTM is only detectable for up to 30 minutes after training onset using this aversive conditioning procedure [25]. Taking these findings into account, we hypothesize that sucrose consumption suppresses the expression of lARM.

Sugar consumption is regulated depending on the satiation state of the animal. In *Drosophila* larvae, hemolymph carbohydrate levels negatively correlate with sucrose consumption [27]. To ensure that this point of high sugar consumption was actually reached in our experiments, we examined the time at which sucrose consumption reached saturation by using a dye-feeding assay [28] (S1A Fig and S1B Fig). During the first 15 and 30 min, a steady increase in sucrose consumption was observed (S1A Fig and S1B Fig, S1 Table). By contrast, larvae feeding for 60 min showed sucrose ingestion behavior that was similar to that of larvae feeding on a dye-only solution (S1A Fig and S1B Fig, S1 Table), indicating that sucrose consumption had reached saturation within 60 min and both groups ingested the same amount of dye. Next, we confirmed that task-relevant sensory-motor abilities like naïve odor preference and salt avoidance were not altered after sucrose consumption (S1C Fig, S1 Table and S2 Table). Strikingly, the suppression of lARM after caloric intake was specific for sucrose and was an immediate effect, as neither the consumption of yeast for 60 min nor of high-caloric food for 1 day led to a suppression of lARM (S2A Fig and S2B Fig, S1 Table and S3 Table). This suggests the involvement of a fast-acting, specific sugar-detecting mechanism, rather than a general mechanism that monitors overall caloric food intake.

### Sucrose consumption gates a cAMP-dependent memory and inactivates *radish*-dependent lARM

**Fig 2:**
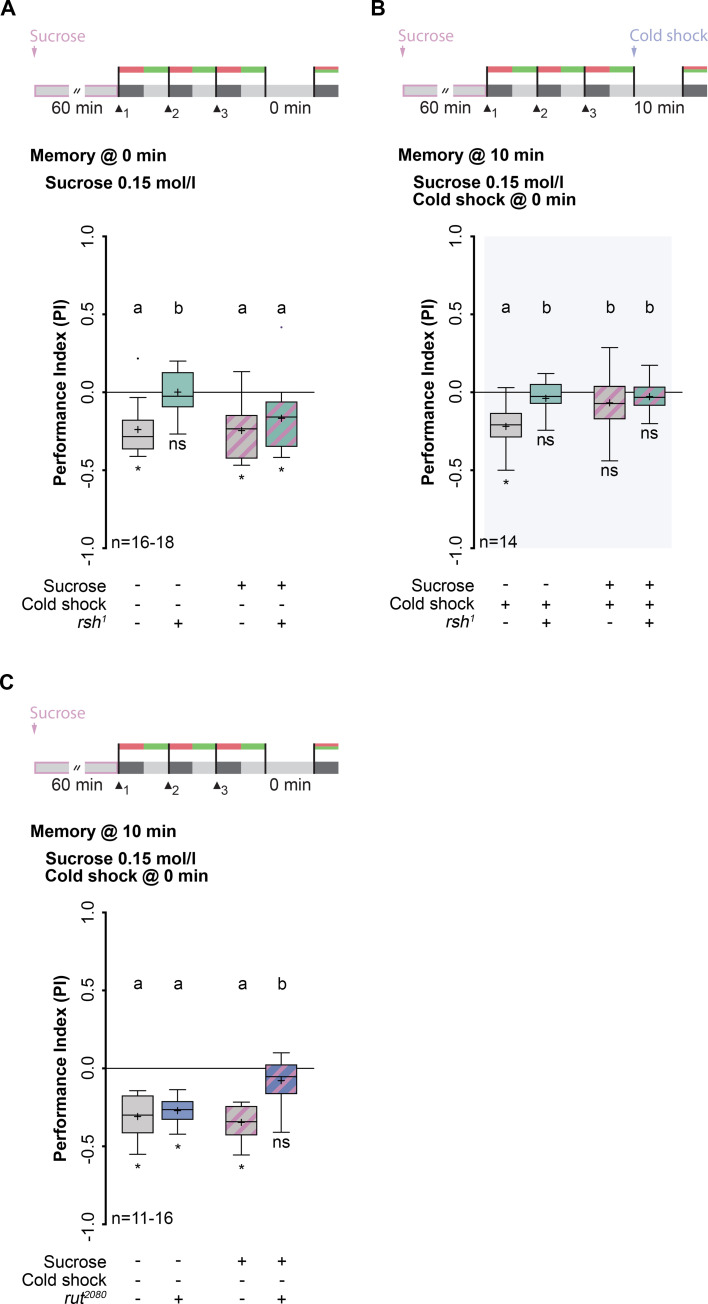
Sucrose consumption gates a cAMP-dependent memory and inactivates *radish*-dependent lARM. (A) Top: Training and treatment protocol. Memory was tested directly after training. Bottom: After sucrose consumption, memory formation is no longer impaired in *rsh*^*1*^ mutants. (B) Top: Training and treatment protocol. Cold shock was applied to all groups. Memory was tested 10 min after training. Bottom: Memory in *rsh*^*1*^ mutants after sucrose consumption is sensitive to cold shock treatment since they showed a complete memory loss. (C) Top: Training and treatment protocol. Memory was tested directly after training. Bottom: Sucrose consumption causes memory loss in *rut*^*2080*^ mutants. Wild-type larvae fed either on tap water or sucrose and *rut*^*2080*^ mutant larvae fed only on tap water showed memory formation indistinguishable from each other. Memory performance above the level of chance was tested using Bonferroni-corrected one-sample t-tests or Wilcoxon signed-rank test (ns p≥0.05/4; * p<0.05/4; adjusted significance level α). Differences between groups were determined using two-way ANOVA followed by Bonferroni *post-hoc* pairwise comparisons. Lowercase letters indicate differences between groups (p<0.05). For more statistical details see also S1 Table and S3 Table. Data are shown as Tukey box plots; line, median; cross, mean; box, 75th-25th percentiles; whiskers, 1.5 interquartile range; small circles, outlier (n≥8).

The *radish* (*rsh*) gene [29] plays a pivotal role in the formation of lARM [25]. Using this mutant provides a tool to test whether the memory phase affected by sucrose consumption is equivalent to the molecularly defined lARM. In line with the key role of *rsh* in lARM formation [25], *rsh*^*1*^ mutant larvae that fed on tap water for 60 min showed complete abolishment of an aversive olfactory memory tested directly after training, in contrast to wild-type animals (Fig 2A; S1 Table). However, the aversive olfactory memory of *rsh*^*1*^ mutant larvae that consumed sucrose for 60 min prior to training revealed no significant defect in comparison with wild-type larvae that consumed either tap water or sucrose (Fig 2A; S3 Table). This finding suggests that the memory deficit in this ARM-specific memory mutant can be rescued by sucrose consumption. This further supports our hypothesis that lARM is replaced by an additional memory phase, if the energy state of the animal is sufficient. Next, we analyzed whether this rescue of memory in *rsh*^*1*^ mutants is due to the direct action of sucrose in *rsh*-associated molecular pathways, and the observed aversive olfactory memory is still lARM. Again, we fed *rsh*^*1*^ mutant larvae sucrose for 60 min, followed by conditioning and tested, if the formed aversive olfactory memory in these mutant larvae was sensitive to anesthesia induced by cold shock treatment (Fig 2B). No memory was detectable, indicating that the aversive olfactory memory formed in *rsh*^*1*^ mutants after sucrose consumption was sensitive to cold shock treatment (Fig 2B, S1 Table).

Apparently, sugar consumption induces a memory phase that differs from lARM at the molecular level. Interestingly, previously reported genetic dissections of larval memory revealed that aversive lSTM and lARM utilize different molecular pathways [25,30,31], in which lSTM depends on proper cAMP-induced signaling. Therefore, we tested whether the formation of the cold shock-sensitive memory after sucrose consumption depends on cAMP signaling. We fed the classical learning mutant *rutabaga*^*2080*^ (*rut*^*2080*^), which exhibits the inability to appropriately increase intracellular cAMP level [32], sucrose for 60 min followed by conditioning (Fig 2C). Directly after training, *rut*^*2080*^ larvae fed on tap water showed intact aversive olfactory memory (Fig 2C, S1 Table and S3 Table), in line with the finding that *rsh*-dependent lARM, but not cAMP-dependent lSTM, is prevalent at this time point [25]. However, aversive olfactory memory after sucrose consumption in *rut*^*2080*^ mutants was completely abolished (Fig 2C, S1 Table). These findings indicate that the newly formed aversive olfactory memory, induced through sucrose consumption, replaces *rsh*-dependent lARM with a *rut*-dependent memory. Therefore, sugar consumption triggers a switch between molecular pathways determining memory phases.

### Activity of the insulin receptor is necessary for suppression of lARM after sucrose consumption

**Fig 3:**
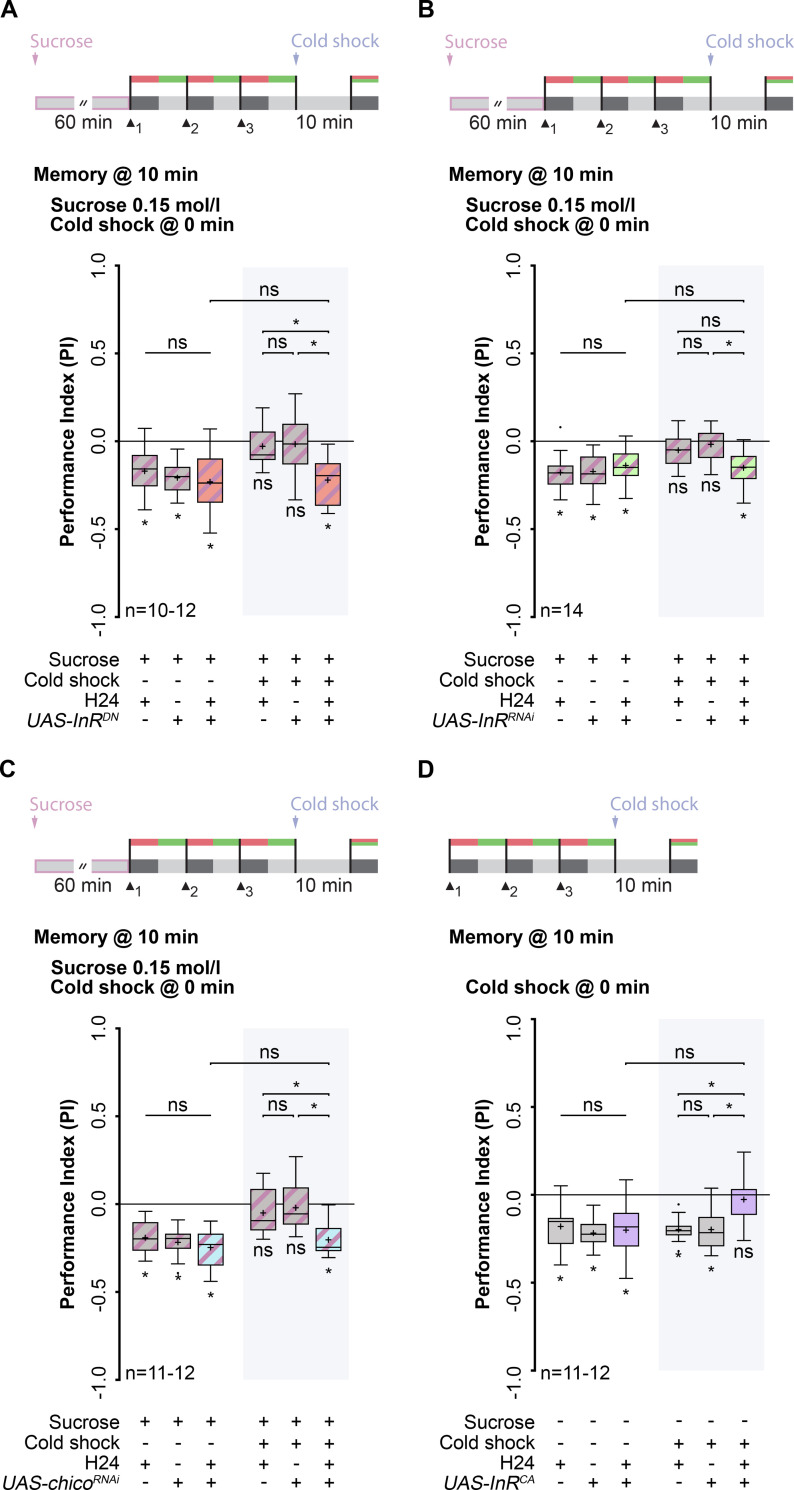
Activity of the insulin receptor is necessary for suppression of lARM after sucrose consumption. (A) Top: Training and treatment protocols. All groups consumed sucrose for 60 min and identification of lARM was carried out by applying a cold shock directly after training. Memory was tested 10 min after training. Bottom: Expression of a dominant negative form of the insulin receptor (UAS-*InR*^*DN*^) in KCs using the driver line H24 prevents the suppression of lARM formation triggered by sucrose consumption. (B) Top: Training and treatment protocols. All groups consumed sucrose for 60 min and identification of lARM was carried out by applying a cold shock directly after training. Memory was tested 10 min after training. Bottom: Knockdown of the insulin receptor by driving expression of UAS-*InR*^*RNAi*^ via H24 prevents the suppression of lARM formation triggered by sucrose consumption. (C) Top: Training and treatment protocols. All groups consumed sucrose for 60 min and identification of lARM was carried out by applying a cold shock directly after training. Memory was tested 10 min after training. Bottom: Knockdown of the insulin receptor substrate (IRS) by driving expression of UAS-*chico*^*RNAi*^ via H24 prevents the suppression of lARM formation triggered by sucrose consumption. (D) Top: Training and treatment protocols. Identification of lARM was carried out by applying a cold shock directly after training. Memory was tested 10 min after training. Bottom: Expression of a constitutively active form of the insulin receptor (UAS-*InR*^*CA*^) in KCs using the driver line H24 leads to the formation of a cold shock sensitive memory since larvae showed a complete loss of memory. Memory performance above the level of chance was tested using Bonferroni-corrected one-sample t-tests (ns p≥0.05/6; * p<0.05/6; adjusted significance level α). Differences between the groups were determined using two-way ANOVA followed by Bonferroni *post-hoc* pairwise comparisons and are depicted above the respective box plots (ns p≥0.05; * p<0.05). For more statistical details see also S1 Table and S3 Table. Data are shown as Tukey box plots; line, median; cross, mean; box, 75th-25th percentiles; whiskers, 1.5 interquartile range; small circles, outlier (n≥8).

In *Drosophila*, the insulin-like growth factor signaling (IIS) pathway is not only essential for maintaining energy storage and glucose metabolism, but also for regulating lifespan and aging, reproduction, nutrient sensing, and cellular growth [33]. In contrast to mammals, which have a large family of IIS-receptors, *Drosophila* has only one insulin receptor (DInR), but eight insulin-like peptides [34,35]. It has been shown that the DInR is necessary for the formation of aversive olfactory LTM in adult *Drosophila* and for the formation of intermediate-term memory in aged flies [36,37]. DInR is strongly expressed in the larval central nervous system (CNS), as well as that of adults [38]. Furthermore, mapping the developmental expression atlas of genes in MB neurons revealed an expression of the DInR in the MB of *Drosophila* third instar larvae [39]. This is important because the synapses that change in the course of associative olfactory learning and thereby mediate memory formation could be localized to the MB KCs, both in adult and larval *Drosophila* [24,40]. Thus, we tested whether the suppression of lARM after sucrose consumption depends on proper insulin signaling in the MB of larval *Drosophila*. First, we expressed a dominant negative variant of the DInR (UAS-*InR*^*DN*^) in all KCs using the driver line OK107. We fed OK107/UAS-*InR*^*DN*^ and both control groups (OK107/+ and UAS-*InR*^*DN*^/+) sucrose for 60 min followed by conditioning and cold shock treatment (S3A Fig). Both control groups receiving cold shock treatment showed a complete abolishment of aversive olfactory memory after sucrose consumption (Fig 3A, S1 Table). By contrast, larvae expressing *InR*^*DN*^ in KCs (OK107/UAS-*InR*^*DN*^) showed an intact aversive olfactory memory comparable to that of the three genetic groups (OK107/+, UAS-*InR*^*DN*^/+ and OK107/UAS-*InR*^*DN*^) that did not receive any cold shock after conditioning (S3A Fig, S1 Table and S3 Table). All task-relevant sensory-motor abilities were unaltered after sucrose consumption (S3B Fig, S1 Table and S3 Table); however, larvae expressing *InR*^*DN*^ in KCs (OK107/UAS-*InR*^*DN*^) showed a slight reduction in sucrose consumption (S3C Fig, S1 Table).

Although this is in line with the observation that inhibition of insulin signaling in the neurons of the MB reduces food intake [41], we cannot exclude side effects of using OK107 to express the dominant negative variant of the DInR in the KCs. Besides expression of OK107 in all larval KCs it exhibits also a limited expression in the ventral nerve cord (VNC) and in insulin producing cells (IPCs) [25,42–44]. Therefore, we repeated the experiment with a second mushroom body specific H24 driver line. This driver line labels also the entire set of larval KCs but the expression profile is nearly specific [25,42,45]. Indeed, expressing the dominant negative variant of the DInR (UAS-*InR*^*DN*^) using H24 had the same effect on memory formation after the consumption of sucrose as OK107 had (Fig 3A). All task-relevant sensory-motor abilities were unaltered after sucrose consumption (S4A Fig, S1 Table and S4 Table). Since sucrose consumption is not affected in larvae expressing the dominant negative variant of the DInR in KCs using H24 (H24/UAS-*InR*^*DN*^) (S5A Fig, S1 Table), we concluded that the presence of lARM in these larvae is due to the dominant negative activity of the DInR and not due to a reduction in sucrose consumption. Additionally, a specific RNAi knockdown of the DInR using UAS-*InR*^*RNAi*^ and H24 confirmed the initial finding that the activity of the insulin receptor is necessary for the suppression of lARM after sucrose consumption (Fig 3B, S1 Table and S3 Table). All task-relevant sensory-motor abilities were unaltered after sucrose consumption in H24/UAS-*InR*^*RNAi*^ larvae (S4B Fig, S1 Table and S4 Table) and consumption is not affected (S5B Fig, S1 Table). Second, in *Drosophila* the dInR is auto-phosphorylated upon activation and subsequently phosphorylates the SHB2 domain-containing insulin receptor substrate (IRS) homolog Chico [46,47]. To analyse the extent of proper insulin signaling playing a role in suppressing lARM after sucrose consumption, we specifically downregulated the IRS homolog Chico in KCs using UAS-*chico*^*RNAi*^ and the driver line H24. We fed H24/UAS-*chico*^*RNAi*^ and both control groups sucrose for 60 min followed by conditioning and cold shock treatment (Fig 3C). In line with the previous findings, larvae with a knockdown of Chico in KCs showed an intact aversive olfactory memory comparable of that of the three genetic groups that did not receive any cold shock after conditioning (Fig 3C, S1 Table and S3 Table). Again, both control groups showed a complete abolishment of aversive olfactory memory after sucrose consumption (Fig 3C, S1 Table). All task-relevant sensory-motor abilities were unaltered after sucrose consumption (S4C Fig, S1 Table and S4 Table) and consumption is not affected (S5C Fig, S1 Table). Third, to determine if insulin signaling is sufficient to induce suppression of lARM, we expressed a constitutively active variant of the DInR (UAS-*InR*^*CA*^) in all KCs using the driver line H24. Note here, no group were fed with sucrose prior to training (Fig 3D). Now, larvae expressing (UAS-*InR*^*CA*^) in KCs showed a complete abolishment of aversive olfactory memory after cold shock treatment (Fig 3D, S1 Table), whereas both control groups showed aversive olfactory memory comparable to that of the three genetic groups (OK107/+, UAS-*InR*^*CA*^/+ and OK107/UAS-*InR*^*CA*^) that did not receive any cold shock after conditioning (Fig 3D, S1 Table and S3 Table). All task-relevant sensory-motor abilities were unaltered between the genetic groups (S4D Fig, S1 Table and S4 Table). Consequently, we conclude that intact insulin signaling is necessary for the suppression of lARM and for the observed switch in memory phases after sucrose consumption. Together with the results demonstrating that a constitutively active variant of the DInR promotes the suppression of lARM after odor-high salt conditioning, we conclude that DInR limits the expression of lARM and gates the expression of a cold shock sensitive memory component.

### Rapid consolidation of a *de-novo* protein synthesis dependent memory after the consumption of sucrose

**Fig 4:**
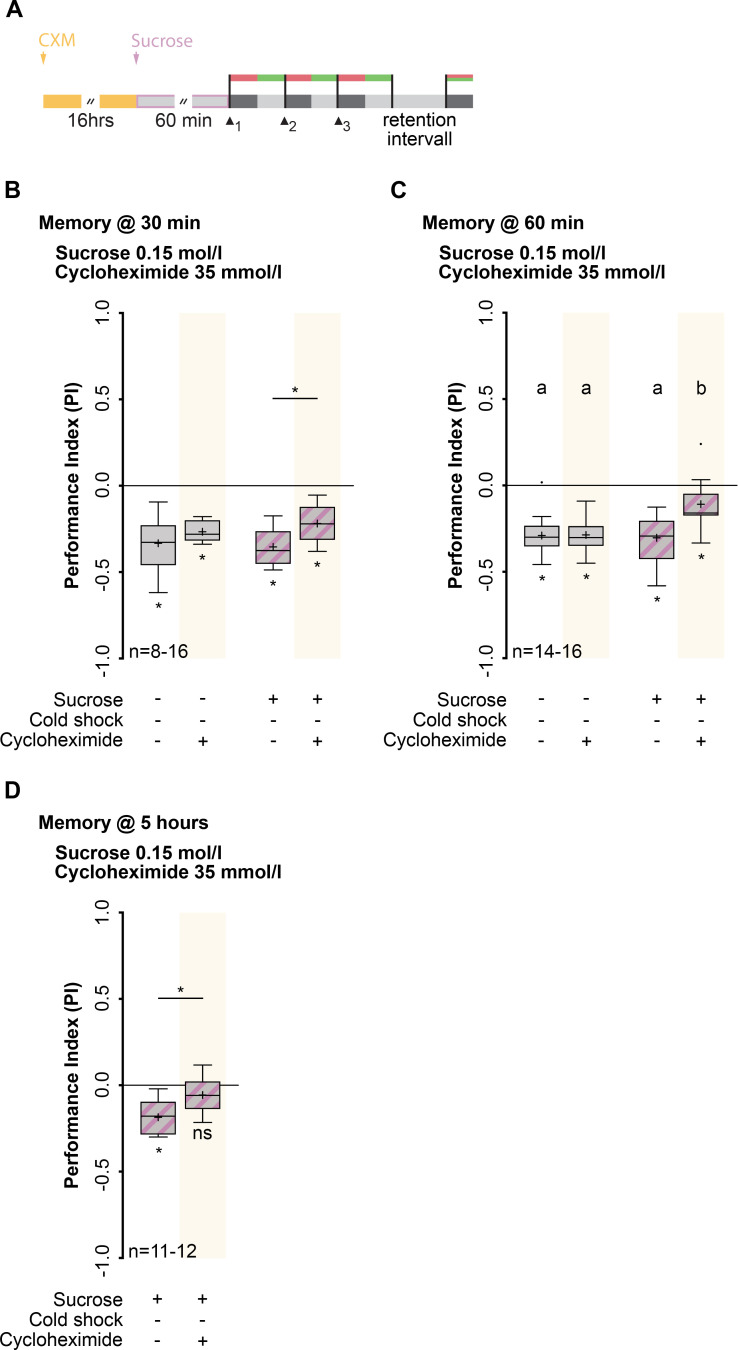
Rapid consolidation of a *de-novo* protein synthesis dependent memory after the consumption of sucrose. (A) Training and treatment protocols. Before feeding on sucrose, larvae were fed for 16 hours on cycloheximide (CXM). Memory was tested at different time points after training. (B) Memory tested 30 min after training was only statistically different between larvae that consumed sucrose with or without CXM treatment. (C) Memory tested 60 min after training larvae that consumed sucrose showed only a slight memory after CXM treatment. This memory was statistically significant different to all other groups of larvae. However, it was not completely abolished. (D) The memory formed after sucrose consumption is stable up to 5 hours and is completely abolished in larvae consuming CXM prior to training. For (B) and (C) memory performance above the level of chance was tested using Bonferroni-corrected one-sample t-tests (ns p≥0.05/4; * p<0.05/4; adjusted significance level α). Differences between groups were determined using two-way ANOVA followed by Bonferroni *post-hoc* pairwise comparisons and are depicted above the respective box plots. In (B) * indicates p<0.05. In (C) lowercase letters indicate differences between groups (p<0.05). For (D) memory performance above the level of chance was tested using Bonferroni-corrected one-sample t-test (ns p≥0.05/2; * p<0.05/2; adjusted significance level α). Differences between groups were determined using unpaired t-test and are depicted above the respective box plots; * indicates p<0.05). For more statistical details see also S1 Table, S2 Table and S3 Table. Data are shown as Tukey box plots; line, median; cross, mean; box, 75th-25th percentiles; whiskers, 1.5 interquartile range; small circles, outlier (n≥8).

We have shown, that after sucrose consumption lARM is suppressed and a second, cAMP dependent memory component is formed (Figs 1B and 2C). But which memory phase, exactly, is induced through sucrose consumption? It was shown that rut-dependent lSTM after odor-high salt conditioning is only detectable for up to 30 minutes after training onset and not visible in longer lasting protocols using two or three cycles [25]. Therefore, we first determined whether the memory was stable over a longer period of time. We fed wild-type larvae sucrose for 60 min prior to conditioning and tested the memory 60 min after training (S4A Fig). The observed olfactory memory was found to be more stable than lSTM, based on the fact that it was still detectable after 60 min and was as robust as lARM formed without sucrose feeding (S4A Fig, S1 Table and S2 Table). It was shown that larval *Drosophila* fed with sucrose prior to electric shock olfactory conditioning form a stable adult memory through metamorphosis [48]. Therefore, we were wondering if feeding on sucrose can also induce a stable, *de-novo* protein synthesis dependent memory after odor-high salt conditioning. Consequently, we tested whether the memory formed after sucrose consumption was dependent on *de-novo* protein synthesis by feeding larvae with the translation-inhibitor cycloheximide (CXM) for 16 hours before the sucrose feeding [18,25] (Fig 4A) and tested the memory at several time points after training (Fig 4B–4D). Surprisingly, wild-type larvae fed on sucrose showed a memory detectable at 30 min, 60 min and even at 5 hours after training (Fig 4B–4D, S1 Table). Therefore, we concluded that the newly formed memory was long-lasting on a larval time scale. Furthermore, the formed memory becomes gradually dependent on *de-novo* protein synthesis since wild-type larvae treated with CXM and fed sucrose showed a statistically significant decrease in olfactory aversive memory tested at 30 min, 60 min and 5 hours after conditioning when compared to control groups, with the effect being the strongest at 5 hours (Fig 4B–4D, S1 Table, S2 Table and S3 Table). At this time point the memory was completely abolished (Fig 4D, S1 Table and S2 Table). Please note controlling for CXM treatment tested at 5 hours is not possible since it was shown that lARM after odor-high salt conditioning is only visible up to 4 hours [25]. Next, we tested if the observed sucrose induced memory is also dependent on functional modifications of relevant molecular signaling pathways within the MB of Drosophila larvae. It is widely accepted that the transcription factor cAMP response element-binding protein (CREB) is universally required for LTM in adult *Drosophila* and represents an evolutionary conserved molecular inducer for LTM [49]. Expression of a repressor isoform of *Drosophila* CREB (dCREB2-b) via H24 specifically in the MB KCs reduces sucrose induced 5 hours memory significantly (S6D Fig, S4 Table). These findings indicate that sucrose consumption leads to the suppression of lARM and, instead, promotes a rapid consolidation of a *de-novo* protein synthesis dependent (PSD) memory, which is also reflected in the dependency of the activity of the CREB transcription factor. Both the dependence of memory formation on de-novo and on CREB activity fulfil the commonly used defining criteria for LTM [49].

Typically, in *Drosophila* adults a *de-novo* protein synthesis dependent longer lasting memory is induced by multiple training trials that are separated by temporal spaces [18]. We tested whether the sugar-induced formation of PSD memory shown here matched the time course of spaced training-induced PSD memory. After three spaced training trials, no PSD memory was observed (S4B Fig, S1 Table and S2 Table). This result was in contrast to sugar-promoted PSD memory formation, shown here to be inducible even after only three training trials. Therefore, we postulate that sugar gates PSD memory formation independent of the requirement of temporal spaces. However, it has been shown that blocking protein synthesis using CXM has a deleterious effect over a longer period of time; specifically, larvae do not properly pupate or enclose [25]. Therefore, we tested whether sucrose consumption after CXM treatment was impaired by feeding larvae CXM for 16 hours. Larvae consumed a detectable amount of liquid dye (0.091±0.025 μl/larva/h, one-sample t-test, p = 0.002) (S2 Data) and the consumption of sucrose was not altered after CXM treatment (S4C Fig, S1 Table). Therefore, the effect of CXM on memory formation cannot be attributed to impaired sucrose consumption.

## Discussion

Establishing a memory requires the timely controlled action of different neuronal circuits, neurotransmitters, neuromodulators and molecules. It is known that after classical aversive olfactory conditioning, *Drosophila* adults form two mutually exclusive longer lasting memory types—LTM and ARM—which can be distinguished based on their dependency on *de-novo* protein synthesis [18,19,50]. The occurrence of such genetically and functionally distinct memory phases is conserved in the animal kingdom, shown in honeybees, in *Aplysia*, and also in vertebrates [51–54].

The hypothesis that protein synthesis-dependent LTM formation is energetically costly and, therefore, restricted to favorable nutritional conditions, is based on a study of adult *Drosophila* [15]. Furthermore, after a spaced training protocol known to induce LTM formation [18,55], flies increased their sugar consumption [20]. Therefore, it seems that the formation of LTM is closely related to energy metabolism, such that the cost of this process must be compensated with increased sugar consumption. Larval *Drosophila* undergoes metamorphosis and the accumulated energy storage during this stage contributes to somatic maintenance and reproduction in adults [56]. Therefore, these larvae present a model system in which the energetic cost of LTM formation far exceeds the potential benefit, especially considering that this memory faces potential degradation during metamorphosis.

**Fig 5:**
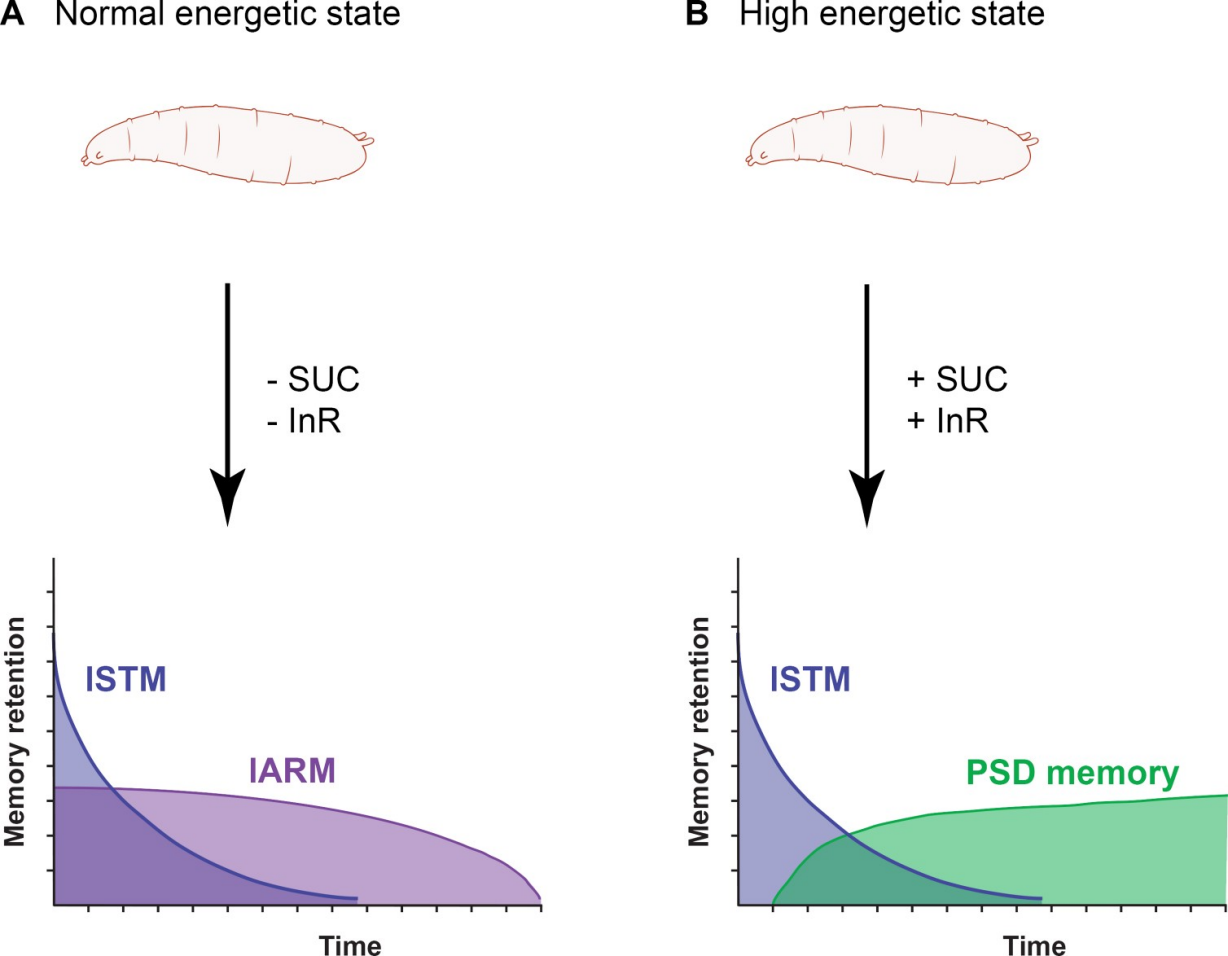
Working hypothesis on the state-dependent switch between memory components after elevating the energetic state of *Drosophila* larvae. (A) In the absence of sucrose or by downregulation of the InR in the MB KCs *Drosophila* larvae form lSTM and lARM. (B) By feeding sucrose prior to conditioning a sugar-promoted PSD memory instead of lARM is triggered, and this is dependent on the activity of the InR in the MB KCs.

Seen in this light, and along with fact that larvae possess all the necessary cellular machinery, we hypothesized that short-term feeding on sucrose directly before training could result in a surplus of energy such that LTM formation is induced instead of ARM. Indeed, we show here that feeding larvae sugar before conditioning is also sufficient to trigger a switch between two consolidated memory phases, even with a less intensive training protocol. This implies that LTM formation is based on two gating mechanisms: one responding to the training intensity (e.g., temporal spacing of multiple trials) and one to the metabolic state. Regarding the first, two slow oscillating dopaminergic neurons have been proposed to act as a gating mechanism for LTM formation at the cost of inhibiting protein synthesis-independent ARM [21]. Regarding the latter, we propose a mechanism in the brain of larval *Drosophila* that directly senses the metabolic state at the time of training and is furthermore independent of the training regime **(Fig 5)**. Without feeding on sucrose or by knocking down the InR in the MB KCs, two co-existing memory phases are visible after aversive olfactory conditioning (lSTM and lARM, Fig 5A). However, by elevating the energetic state by feeding sucrose and through an insulin-signaling-dependent gating mechanism, the *rsh*-dependent lARM is suppressed and a cAMP-dependent protein synthesis dependent longer lasting memory is visible (Fig 5B). One general feature of LTM formation across species is that initially it exists as a labile state and can be disrupted very easily. However, with time passing by it is becomes stronger and resistant to disruption, or in other words, consolidated. One hallmark of this consolidation process is the initiation of a *de-novo* protein synthesis phase. Even though the sucrose-induced memory is becoming sensitive to CXM treatment relatively shortly after training (Fig 5B), it is still dependent on the transcription factor CREB (S6D Fig), which is a characteristic of true LTM [49]. This supports the finding that *Drosophila* larvae can form a CREB-dependent memory [25]. Therefore, we have determined that the conserved principal of cAMP-dependent, protein synthesis-dependent longer lasting memory formation holds true also for *Drosophila* larvae, although they undergo metamorphosis and most likely all formed longer lasting memory components are erased after the re-structuring of brain connectivity in the course of pupation. Although, we have shown here the existence of a protein synthesis dependent longer lasting memory, it remains still elusive, if *Drosophila* larvae are capable to form LTM comparable with the adult stage.

Remarkably, we have also demonstrated a potential gating mechanism underlying the formation of LTM. Previous work has shown that LTM in adult *Drosophila* leads to a subsequent increase in energy metabolism. We take this a step further by demonstrating that increasing the energetic state of larvae before the training begins is sufficient to trigger the formation of protein synthesis dependent longer lasting memory even after a less intense training protocol. This means that, although a protein synthesis dependent longer lasting memory is highly costly (and in the case of larvae theoretically redundant), its formation can be forced under the right circumstances due to the presence of a mechanism for the detection of energetic surplus that negates this high cost. This is also in agreement with recent studies showing that glycolytic enzymes are required in the MB of adult *Drosophila* for the formation of aversive olfactory memory [57]. This means that the brain of larval *Drosophila*—and potentially brains of other animals as well [58]—is not only a calculation device to decide if incoming sensory information is of importance, for example in the case of repetitions of the same stimulus, but can also sense and balance existing resources and decide if forming an expensive memory is an affordable or life-threatening luxury, especially for larvae whose main behavioral activity is taking in food.

Sugar by itself is assumed to be a primary source of energy, with circulating sugar levels reflecting the energetic state of an animal. Controlling the metabolic homeostasis is regulated via the *Drosophila* orthologs of glucagon (adipokinetic hormone, AKH) and insulin (*Drosophila* insulin-like peptide, DILP) [59]. Both have been proven to be involved in feeding and foraging behaviors and are controlled contrastingly through glucose [60–62]. Additionally, it has been shown that the InR is acutely required for LTM formation in *Drosophila* adults [37]. Strikingly, we show here that both increase in energetic state and insulin signaling are necessary to mediate the formation of a protein synthesis dependent longer lasting memory in *Drosophila* larvae (Fig 5B). We concluded that insulin signaling in the MB KCs of *Drosophila* larvae can directly sense the elevated energetic state provoked by feeding sucrose directly before training and as a result mediate the state-dependent switch between lARM and a protein synthesis dependent memory. Beyond that, the involvement of insulin signaling in memory formation has striking parallels in mammals as well. For example, downregulation of an insulin receptor in the hippocampus of mice leads to spatial learning deficits [63]. Moreover, injections of insulin reversed memory deficits caused by Alzheimer’s disease, and in stroke patients an intranasal insulin treatment has been shown to improve hippocampal-dependent declarative memory in healthy humans [64]. Given the fact that the molecular underpinnings of both memory formation and insulin signaling are highly conserved across the animal kingdom [65,66], this correspondence among taxa is not surprising. Rather, it corroborates the general validity of model organisms like *Drosophila*. Thus, our finding that insulin signaling gates the formation of a protein synthesis dependent longer lasting memory and inhibits an alternative memory component could be of importance for the study in higher organisms, including humans.

## Material and methods

### Fly stocks

Fly strains were reared on standard *Drosophila* medium at 25°C with 70% humidity in a 12-hour light-dark cycle. Crosses were raised at 18°C or 25°C with 70% relative humidity in a 12-hour light-dark cycle on standard *Drosophil*a medium. Flies were transferred to new vials and allowed to lay eggs for 2 days. For all experiments, 6-day-old foraging (feeding) third instar larvae were used. The wild-type strain was *Canton-S* (denoted here as wild-type). We used the learning mutants *rut*^*2080*^ (obtained from the Bloomington Drosophila Stock Center, BDSC No.: 9405) and *rsh*^*1*^ (kindly provided by T. Preat) [29,32]. All lines were outcrossed over several generations with wild-type *Canton-S* that was used as a genetic control. To express Gal4 in all larval Kenyon cells (KCs) we used the driver line OK107 [42,43] (obtained from the Bloomington Drosophila Stock Center, BDSC no.: 106098) and H24 [42,45] (obtained from the Bloomington Drosophila Stock Center, BDSC no.: 51632). The effector lines UAS-*dInR*^*A1409K*^ (denoted here as UAS-*dInR*^*DN*^) (obtained from the Bloomington Drosophila Stock Center, BDSC No.: 8253), UAS-*dInR*^*RNAi*^ (obtained from the Vienna Drosophila RNAi Centre, VDRC no.: 992), UAS-*chico*^*JF02964*^ (denoted here as UAS-*chico*^*RNAi*^) (obtained from the Bloomington Drosophila Stock Center, BDSC no.: 28329) and UAS-*dInR*^*del*^ (denoted here as UAS-*dInR*^*CA*^) (obtained from the Bloomington Drosophila Stock Center, BDSC no.: 8248) was used to manipulate insulin signaling within the KCs. The UAS-*dInR*^*DN*^ transgene carries an amino acid replacement in the kinase domain (K1409A) of the *Drosophila* insulin receptor (dInR), which results in its dominant negative activity [67]. The UAS-*dInR*^*CA*^ transgene contains a deletion of most of the alpha subunit, which results in a constitutively active form of the InR. The UAS-*dCreb2-b* transgene is a repressor isoform of *Drosophila* CREB (CREB2b) (kindly provided by A. Thum) [68].

### Aversive olfactory learning and memory

Aversive olfactory learning and memory was performed at 23°C under standard laboratory conditions. Standard aversive olfactory conditioning experiments were performed using an odor-high salt conditioning paradigm, as previously described [25]. Experiments were conducted on assay plates (92-mm diameter, Sarstedt, Nümbrecht, cat. no.: 82.1472) filled with a thin layer of 2.5% agarose containing either pure agarose (Sigma Aldrich, cat. no.: A5093, CAS no.: 9012-36-6) or agarose plus 1.5 M sodium chloride (Sigma Aldrich, cat. no.: S7653, CAS no.: 7647-14-5) [25,26]. As olfactory stimuli, we used 10 μl amyl acetate (AM, Sigma Aldrich cat. no.: 109584; CAS No.: 628-63-7; diluted 1:250 in paraffin oil, Sigma Aldrich cat. no.: 18512, CAS no.: 8012-95-1) and benzaldehyde (BA, undiluted; Sigma Aldrich cat. no.: 418099, CAS no.: 100-52-7). Odorants were loaded into custom-made Teflon containers (4.5-mm diameter) with perforated lids [69]. Learning ability was tested by exposing a first group of 30 larvae to AM while they crawled on agarose medium that additionally contained sodium chloride as a negative reinforcer. After 5 min, the larvae were transferred to a fresh Petri dish in which they were allowed to crawl on a pure agarose medium for 5 min while being exposed to BA (AM+/BA). A second group of larvae received the reciprocal training (AM/BA+). Three training cycles were conducted. To test the memory after training, larvae were transferred onto another agarose plate and kept there for the indicated time before the memory was tested. To increase the humidity, tap water was added. Memory was tested by transferring larvae onto fresh agarose plates containing 1.5 M sodium chloride, on which AM and BA were presented on opposite sides. After 5 min, individuals located on the AM side (#AM), BA side (#BA), or in a 1-cm neutral zone were counted. We determined a preference index for each training group by subtracting the number of larvae on the BA side from the number of larvae on the AM side, and dividing by the total number of counted individuals (#TOTAL), as follows:

$$PREF_{AM+/BA}=\left(\#AM‐\#BA\right)/\#TOTAL$$ (1A)

$$PREF_{AM/BA+}=\left(\#AM‐\#BA\right)/\#TOTAL$$ (1B)

To specifically measure the effect of associative learning that is of the odor-reinforcement contingency, we then calculated the associative Performance Index (PI) as the difference in preference between the reciprocally trained larvae, as follows:

$$PI=\left(PREF_{AM+/BA}–PREF_{AM/BA+}\right)/2$$ (2)

Negative PIs represented aversive associative learning, whereas positive PIs indicated appetitive associative learning. Division by 2 ensured that the scores were bounded between -1 and 1).

### Manipulation of the nutritional state

The nutritional state of larvae was manipulated by feeding 0.15 M sucrose (Sigma Aldrich, cat. no.: 84097, CAS no.: 57-50-1) for 60 min. A group of 30 larvae were either fed with 0.15 M sucrose mixed with tap water (+SUC) or with tap water (-SUC, control group). Larvae were placed in a Petri dish (35-mm diameter, Sarstedt, Nümbrecht, cat. no.: 82.1135.500) containing 2.5% agarose, and 1.5 ml of sucrose solution (+SUC) or tap water (-SUC) was added. This volume ensured that larvae did not crawl out of the sucrose solution and additionally prevented them from drowning. The larvae were allowed to feed for 60 min (if not stated otherwise) at 23°C. Zeitgeber time and humidity were kept constant for these experiments. The larvae were washed gently with tap water after being fed and transferred to an empty Petri dish containing 2.5% agarose.

### Quantification of sucrose consumption

To quantify sucrose consumption we used a modified feeding assay, as previously described [28]. A group of 30 larvae were placed in a Petri dish (35-mm diameter, Sarstedt, Nümbrecht, cat. no.: 82.1135.500) containing 2.5% agarose and either 1.5 ml 0.15 M sucrose + 2% indigo carmine (w/vol) (Sigma Aldrich, cat. no.: 57000, CAS no.: 860-22-0) mixed in tap water (+SUC +IC, experimental group), 2% (w/ml) indigo carmine mixed in tap water (+IC, dye-only control) or tap water (-IC, blank control). Again, 1.5 ml of the specific solution ensured that larvae did not crawl out of the solution and additionally prevented them from drowning. The larvae were allowed to feed for 1 hour at 23°C. Zeitgeber time and humidity were kept constant for these experiments. After 60 min, larvae were rinsed with tap water, transferred into 2-ml Eppendorf cups containing 500 μl of 1 M L-ascorbic acid (Sigma Aldrich, cat. no.: A7506, CAS no.: 50-81-7) and bead-based homogenized for 2 min using a Qiagen TissueLyser LT at a frequency of 50/s. After centrifugation at 14,800 rpm for 5 min at 23°C, the supernatant (400 μl) was transferred to Micro Bio-Spin^TM^ Columns (Bio-Rad) and centrifuged again at 14,800 rpm for 5 min at 23°C for filtration. Subsequently, 200 μl of the supernatant was transferred into a new Eppendorf cup (1.5 ml) and centrifuged for a third time at 14,000 rpm for 2 min at 23°C. To quantify sucrose consumption, 100 μl supernatant was transferred to a 96-well plate (Greiner Bio-One, cat. no.: 655061) and absorbance was measured at 610 nm [28] using a BioTek^TM^ Epoch Spectrophotometer. The corrected absorbance ABS (CORR) of each measurement was calculated by subtracting the mean absorbance of 1 M ascorbic acid (ABS_AA_) from the relative absorbance of either the blank control (ABS_-IC_), dye-only control (ABS_+IC_), or experimental group (ABS_+SUC +IC_). The relative consumption of sucrose (R.C.) was deduced by calculating the difference between the corrected mean absorbance of the blank control (ABS_-IC_(CORR)), the corrected mean absorbance of the dye-only control (ABS_+IC_(CORR)) and the relative absorbance of the experimental group (ABS_-IC +SUC_(CORR)):

$$R.C.=\left(ABS_{+IC+SUC}\left(CORR\right)‐ABS_{‐IC}\left(CORR\right)\right)‐\left(ABS_{+IC}\left(CORR\right)‐ABS_{‐IC}\left(CORR\right)\right))/(ABS_{+IC}\left(CORR\right)–\left(ABS_{‐IC}\left(CORR\right)\right)$$ (3)

The blank control and the dye-only control were measured at every experiment and for every genotype on the same day. An R.C. value of 0 indicated that the larvae in the experimental group ate as much as the dye-only control larvae, a R.C.≤0 indicated that the larvae in the experimental group ate less than dye-only control larvae, and an R.C.≥0 indicated that larvae in the experimental group ate more than larvae in the dye-only control. To verify that the amount of ingested dye is represented in a linearly proportional manner, absorbance at 610 nm was measured for 100 μl of ascorbic acid and 2% (w/ml) indigo carmine in a two-fold serial dilution.

### Cold shock treatment

To distinguish between cold shock sensitive and cold shock resistant memory phases, odor-high salt conditioning was followed by a cold shock treatment, as previously described [25]. Briefly, larvae were incubated in ice-cold tap water (4°C) for 1 min. Larvae were allowed to recover for at least 10 min by transferring them onto fresh agarose plates. They started moving within 2 min and were kept on the agarose plates at 23°C until testing.

### Cycloheximide treatment

To test if aversive olfactory memory induced by feeding sucrose prior to training is dependent on *de novo* protein synthesis, larvae were fed cycloheximide (CXM) as previously described [25]. Briefly, larvae were fed either with 35 mM cycloheximide (+CXM; Sigma Aldrich cat. no.: C7698; CAS no.: 66-81-9) or tap water (-CXM, control group) for 16 hours before the experiment. Therefore, 300 μl of CXM solution or tap water was added to the food vials. Before the experiment the larvae were gently washed with tap water and transferred to an empty Petri dish before being fed sucrose and undergoing subsequent odor-high salt conditioning and testing of the aversive olfactory memory at different time points.

### Odor preference and high salt avoidance experiments

To analyze larval olfactory perception, 30 larvae were placed along the midline of a Petri dish containing 2.5% pure agarose, with either a 10 μl amyl acetate- (AM) or a benzaldehyde-containing (BA) odor container on one side and an empty container (EC) on the other side. After 5 min, larvae located on the odor side (#ODOR), the side with the empty container (#EC), or in a 1-cm neutral zone were counted. By subtracting the number of larvae on the odor side from the number of larvae on the EC side, and dividing by the total number of counted individuals (#TOTAL), we determined a preference index for either AM or BA for each training group, as follows:

PREF=(#ODOR‐#EMPTY)/#TOTAL (4)

To investigate high salt avoidance, 30 larvae were placed along the midline of a Petri dish containing pure agarose on one side and agarose plus 1.5 M sodium chloride on the other. After 5 min larvae located on the salt side (#SALT), the agarose side (#AGAROSE), or in a 1-cm neutral zone were counted. By subtracting the number of larvae on the odor side from the number of larvae on the EC side, and dividing by the total number of counted individuals (#TOTAL), we determined a preference index for high salt avoidance for each training group, as follows:

GAI=(#SALT‐#AGAROSE)/#TOTAL (5)

### Quantification and statistical analysis

All statistical analyses and visualizations were conducted with *GraphPad Prism* 8.0.2. Significance level of all statistical test was set to α = 0.05. To compare single groups against the level of chance, we used Bonferroni-corrected two-tailed one-sample t-tests for normally distributed data (Shapiro-Wilk test), otherwise Bonferroni-corrected two-tailed Wilcoxon signed-rank tests; significance level is adjusted to α/n (α = 0.05, n is the number of test). Significance is indicated in all figures below the respective boxplot by: (ns) not significant; (*) p< 0.05/n. For comparison between two groups, which did not violate the assumptions of normality (Shapiro-Wilk test) and homogeneity of variance (Bartlett's test) were analyzed with two-tailed unpaired t-test, otherwise two-tailed Mann-Whitney test. Significance is indicated in all figures above boxplots by: (ns) not significant; (*) p<0.05. For comparison between more than two groups, which did not violate the assumption of normality (Shapiro-Wilk test) and homogeneity of variance (Bartlett's test) were analyzed with one-way ANOVA followed by Tukey HSD post-hoc pairwise comparisons between relevant groups, otherwise Kruskal-Wallis test followed by Dunn’s multiple pairwise comparisons. For statistical tests involving two factors, two-way ANOVAs were applied, followed by planned, pairwise multiple comparisons (Bonferroni *post-hoc* pairwise comparisons); significance is indicated in all figures above boxplots by: lowercase letters indicate differences between groups (p<0.05) or (ns) not significant. Respective statistical tests used, sample sizes, and descriptive statistics can be found in Supplemental S1, S2 and S3 Tables for main figures and Supporting Information figures. Data were presented as Tukey box plots, with 50% of the values being located within the boxes and whiskers representing 1.5 interquartile range. Outsiders were indicated as open circles. The median was indicated as a bold line and the mean as a cross within the box plot. Unless stated otherwise, experiments had a sample size of 16. Figure alignments were performed with *Adobe Photoshop CC* 2019 and *Adobe Illustrator CC* 2019.

## Supporting information

S1 Fig Time-dependent sucrose feeding in wild-type larvae and task-relevant sensory-motor abilities. (A) Top: Sucrose consumption quantification using a photometer-quantified dye feeding assay. Wild-type larvae fed either on dye alone (left) or sucrose + dye (right) for 15, 30, or 60 min. By normalizing to the dye-only group a Relative Consumption of Sucrose Index (R.C.) was calculated. Bottom: Sucrose consumption is dependent on the satiation state of larvae. An increased sucrose consumption was observed at 15 and 30 min, whereas sucrose consumption at 60 min was similar to dye-only group. (B) Representative image showing wild-type larvae fed for 15 min, 30 or 60 min either on dye or on sucrose + dye (from left, in order). (C) Top: Odor preference and high-salt avoidance assays after ingesting sucrose for 60 min. Naïve AM preference left, naïve BA preference middle, salt avoidance right. Olfactory perception was analyzed by calculating an Olfactory Preference Index (PREF). High salt avoidance was analyzed by calculating a Gustatory Avoidance Index (GAI). Bottom: Task-relevant sensory-motor abilities were not altered after sucrose consumption. Sucrose consumption, naïve odor preference, and high salt avoidance above the level of chance was tested using Bonferroni-corrected one-sample t-tests or Wilcoxon signed-rank test (ns p≥0.05/2; * p<0.05/2; adjusted significance level α). Differences between groups in (C) were determined using unpaired t-test or Mann-Whitney test. Statistically non-significant differences between groups (p≥0.05) are indicated as ns. For more statistical details see also S1 Table and S2 Table. Data are shown as Tukey box plots; line, median; cross, mean; box, 75th-25th percentiles; whiskers, 1.5 interquartile range; small circles, outlier (n≥8). AM, n-amyl acetate; BA, benzaldehyde.
(TIF)

S2 Fig Aversive olfactory memory formation after a protein-rich diet. (A) Top: Training and treatment protocols. Larvae fed for 60 min on yeast and identification of lARM was carried out by applying a cold shock directly after training. Memory was tested 10 min after training. Bottom: Feeding on yeast does not inhibit lARM formation in wild-type larvae. (B) Top: Training and treatment protocols. Larvae were kept for one day on protein and carbohydrate rich food (denoted here as PCF), which is characterized by a high proportion of sucrose (150 g/l) and added pork fat (10 g/l). Identification of lARM was carried out by applying a cold shock directly after training. Memory was tested 10 minutes after training. Bottom: Feeding for one day on protein and carbohydrate rich food does not inhibit lARM formation in wild-type larvae. Memory performance above the level of chance was tested using Bonferroni-corrected one-sample t-tests (ns p≥0.05/4; * p<0.05/4; adjusted significance level α). Differences between groups were determined using two-way ANOVA followed by Bonferroni *post-hoc* pairwise comparisons. Statistically non-significant differences between groups (p≥0.05) are indicated as ns. For more statistical details see also S1 Table and S3 Table. Data are shown as Tukey box plots; line, median; cross, mean; box, 75th-25th percentiles; whiskers, 1.5 interquartile range; small circles, outlier (n≥8). lARM, larval anesthesia resistant memory.
(TIF)

S3 Fig Expression of a dominant negative form in the MB KCs of the insulin receptor leads to the suppression of lARM after sucrose consumption. (A) Top: Training and treatment protocols. All groups consumed sucrose for 60 min and identification of lARM was carried out by applying a cold shock directly after training. Memory was tested 10 min after training. Bottom: Expression of the dominant negative form of the insulin receptor (UAS-*InR*^*DN*^) in KCs using the driver line OK107 prevents the suppression of lARM formation triggered by sucrose consumption. (B) Top: Odor preference and high-salt avoidance assays after ingesting sucrose for 60 min. Naïve amyl acetate (AM) left preference left, naïve benzaldehyde (BA) preference middle, salt avoidance right. Olfactory perception was analyzed by calculating an Olfactory Preference Index (PREF). High salt avoidance was analyzed by calculating a Gustatory Avoidance Index (GAI). Bottom: Task-relevant sensory-motor abilities are not altered in larvae expressing a dominant negative form of the insulin receptor (InR^DN^) in the MB KCs via the OK107 driver line. Bottom left: naïve odor preference for AM. Bottom middle: naïve odor preference for BA. Bottom right: naïve avoidance of a high salt concentration. (C) Top: Sucrose consumption quantification using a photometer-quantified dye feeding assay. A Relative Consumption of Sucrose Index (R.C.) was calculated by normalizing to the dye-only control. Bottom: Expression of a dominant negative insulin receptor (*InR*^*DN*^) transgene in MB KCs via OK107 driver line leads to a small reduction in sucrose consumption. Bottom left: Sucrose consumption in OK107/+ control group. Bottom middle: Sucrose consumption in UAS-*InR*^*DN*^/+ control group. Bottom right: Sucrose consumption in OK107/UAS-*InR*^*DN*^ experimental group. For (A) and (B) memory performance, naïve odor preference and high salt avoidance above the level of chance was tested using Bonferroni-corrected one-sample t-test (ns p≥0.05/6; * p<0.05/6; adjusted significance level α). Differences between the groups were determined using two-way ANOVA followed by Bonferroni *post-hoc* pairwise comparisons and are depicted above the respective box plots (ns p≥0.05; * p<0.05; α = 0.05). For (C) sucrose consumption above the level of chance was tested using Bonferroni-corrected one-sample t-tests (ns p≥0.05/2; * p<0.05/2; adjusted significance level α). For more statistical details see also S1 Table and S3 Table. Data are shown as Tukey box plots; line, median; cross, mean; box, 75th-25th percentiles; whiskers, 1.5 interquartile range; small circles, outlier (n≥8). AM, n-amyl acetate; BA, benzaldehyde; DN, dominant negative; InR, insulin receptor; KC, Kenyon cell; lARM, larval anesthesia resistant memory; UAS, upstream activation sequence.
(TIF)

S4 Fig Task-relevant sensory-motor abilities in larvae with altered insulin signaling in mushroom body Kenyon cell. (A) Odor preference and high-salt avoidance assays after ingesting sucrose for 60 min. Naïve amyl acetate (AM) left preference left, naïve benzaldehyde (BA) preference middle, salt avoidance right. Olfactory perception was analyzed by calculating an Olfactory Preference Index (PREF). High salt avoidance was analyzed by calculating a Gustatory Avoidance Index (GAI). (B) Task-relevant sensory-motor abilities are not altered in larvae expressing a dominant negative form of the insulin receptor (InR^DN^) in the MB KCs via the H24 driver line. Left: naïve odor preference for AM. Middle: naïve odor preference for BA. Right: naïve avoidance of a high salt concentration. (C) Task-relevant sensory-motor abilities are not altered in larvae when knocking down insulin receptors in the MB KCs via UAS-*InR*^*RNAi*^ and H24. Left: naïve odor preference for AM. Middle: naïve odor preference for BA. Right: naïve avoidance of a high salt concentration. (D) Task-relevant sensory-motor abilities are not altered in larvae when knocking down the *Drosophila* insulin receptor substrate homologue *chico* in the MB KCs via UAS-*chico*^*RNAi*^ and H24. Left: naïve odor preference for AM. Middle: naïve odor preference for BA. Right: naïve avoidance of a high salt concentration. (E) Task-relevant sensory-motor abilities are not altered in larvae expressing a constitutively active form of the InR in the MB KCs via UAS-*InR*^*CA*^ and H24. Left: naïve odor preference for AM. Middle: naïve odor preference for BA. Right: naïve avoidance of a high salt concentration. Naïve odor preference and high salt avoidance above the level of chance was tested using Bonferroni-corrected one-sample t-test (ns p≥0.05/3; * p<0.05/3; adjusted significance level α). Differences between the groups were determined using one-way ANOVA followed by Tukey *post-hoc* pairwise comparisons and are depicted above the respective box plots; ns indicates p≥0.05). Data are shown as Tukey box plots; line, median; cross, mean; box, 75th-25th percentiles; whiskers, 1.5 interquartile range; small circles, outlier (n≥8). AM, n-amyl acetate; BA, benzaldehyde; CA, constitutively active; DN, dominant negative; InR, insulin receptor; KC, Kenyon cell; MB, mushroom body; UAS, upstream activation sequence.
(TIF)

S5 Fig Sucrose feeding in larvae with altered insulin signaling in mushroom body Kenyon cell. (A) Sucrose consumption quantification using a photometer-quantified dye feeding assay. A Relative Consumption of Sucrose Index (R.C.) was calculated by normalizing to the dye-only control. (B) Expression of a dominant negative insulin receptor (*InR*^*DN*^) transgene in MB KCs via H24 driver line does not change the sucrose consumption at 60 min. Left: Sucrose consumption in OK107/+ control group. Middle: Sucrose consumption in UAS-*InR*^*DN*^/+ control group. Right: Sucrose consumption in OK107/UAS-*InR*^*DN*^ experimental group. (C) Specific knockdown of insulin receptors in MB KCs via UAS-*InR*^*RNAi*^ and H24 does not change sucrose consumption at 60 min. Left: Sucrose consumption in OK107/+ control group. Middle: Sucrose consumption in UAS-*InR*^*RNAi*^/+ control group. Right: Sucrose consumption in OK107/UAS-*InR*^*RNAi*^ experimental group. (D) Specific knockdown of the *Drosophila* insulin receptor substrate homologue *chico* in the MB KCs via UAS-*chico*^*RNAi*^ and H24 does not change sucrose consumption at 60 min. Left: Sucrose consumption in OK107/+ control group. Middle: Sucrose consumption in UAS-*chico*^*RNAi*^/+ control group. Right: Sucrose consumption in OK107/UAS-*chico*^*RNAi*^ experimental group. (E) Expressing of a constitutively active form of the InR in the MB KCs via UAS-*InR*^*CA*^ and H24 does not change sucrose consumption at 60 min. Left: Sucrose consumption in OK107/+ control group. Middle: Sucrose consumption in UAS-*InR*^*CA*^/+ control group. Right: Sucrose consumption in OK107/UAS-*InR*^*CA*^ experimental group. Sucrose consumption above the level of chance was tested using Bonferroni-corrected one-sample t-tests (ns p≥0.05/2; * p<0.05/2; adjusted significance level α). For more statistical details see also S1 Table. Data are shown as Tukey box plots; line, median; cross, mean; box, 75th-25th percentiles; whiskers, 1.5 interquartile range; small circles, outlier (n≥8). CA, constitutively active; DN, dominant negative; InR, insulin receptor; KC, Kenyon cell; MB, mushroom body; UAS, upstream activation sequence.
(TIF)

S6 Fig Stability of aversive olfactory memory after sucrose consumption and formation of aversive olfactory memory after spaced training. (A) Top: Training and different treatment protocols. Memory was tested 60 min after training. Bottom: Memory after sucrose consumption was statistically indistinguishable from lARM formed after being fed on tap water. (B) Top: Training and different treatment protocols. Wild-type larvae were fed CXM for 16 hours. Odor-high salt conditioning was performed using a spaced training protocol consisting of three cycles separated by 15 min rest intervals (open triangle). Memory was tested 60 min after training. Bottom: The application of CXM did not affect the aversive olfactory memory that was formed after a spaced training protocol. (C) Top: Sucrose consumption quantification in wild-type larvae using a photometer-quantified dye feeding assay. A Relative Consumption of Sucrose Index (R.C.) was calculated by normalizing to the dye-only control. Both groups were fed CXM for 16 hours before feeding on sucrose. Bottom: Sucrose consumption is not altered after CXM treatment. (D) Top: Training and treatment protocol. Memory was tested 5 hours after training, Bottom: Expression of a repressor isoform of CREB (UAS-*dCREB2-b*) in KCs using the driver line H24 resulted in a lower sucrose induced 5 hours memory than in both genetic control groups (H24/+ and UAS-*dCREB2-b*/+). For (A), (B) and (C) memory performance and sucrose consumption above the level of chance was tested using Bonferroni-corrected one-sample t-tests (ns p≥0.05/2; * p<0.05/2; adjusted significance level α). For (A) and (B) differences between groups were determined using unpaired t-test. Statistically non-significant differences between groups (p≥0.05) are indicated as ns. For (D) memory performance above the level of chance was tested using Bonferroni-corrected one-sample t-test (ns p≥0.05/3; * p<0.05/3; adjusted significance level α). Differences between the groups were determined using one-way ANOVA followed by Tukey *post-hoc* pairwise comparisons. Lowercase letters indicate differences between groups (p<0.05). For more statistical details see also S1 Table, S2 Table and S3 Table. Data are shown as Tukey box plots; line, median; cross, mean; box, 75th-25th percentiles; whiskers, 1.5 interquartile range; small circles, outlier (n≥8). CXM, cycloheximide; lARM, larval anesthesia resistant memory.
(TIF)

S1 Table Sample size, mean±s.e.m. and statistical details of test against chance level. (DOCX)

S2 Table Statistical details of unpaired t-test or Mann-Whitney test. (DOCX)

S3 Table Statistical details of two-way ANOVA. (DOCX)

S4 Table Statistical details of one-way ANOVA. (DOCX)

S1 Data Quantitative observations underlying all the figures. (XLSX)

S2 Data Relative consumed concentration after feeding on CXM for 16 hours. (XLSX)
