# Supplementary figures and images for: Insulin signaling represents a gating mechanism between different memory phases in Drosophila larvae
Source: PLoS Genet. 2020 Oct 26;16(10):e1009064. doi: 10.1371/journal.pgen.1009064 (PMC7644093; doi:10.1371/journal.pgen.1009064)

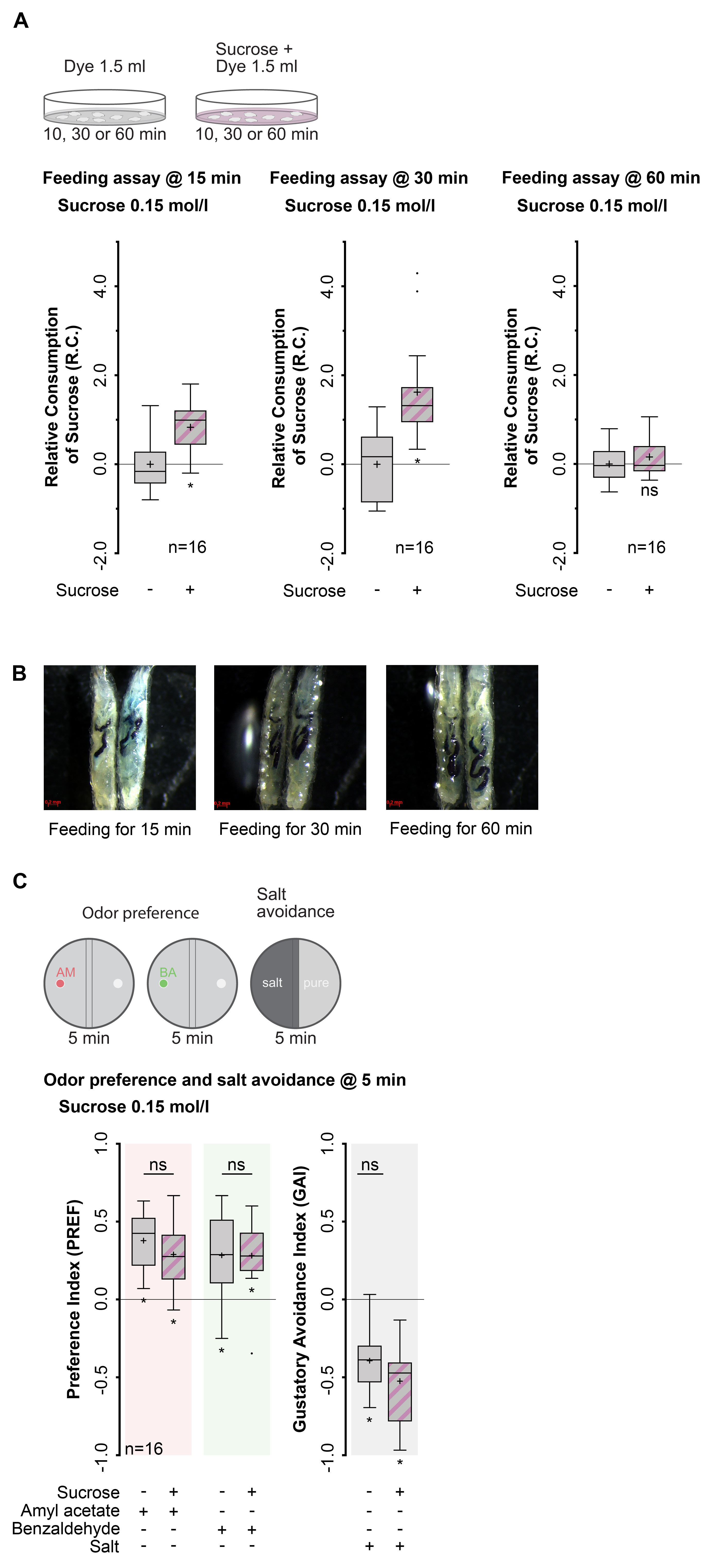

Supplement: S1 Fig — (A) Top: Sucrose consumption quantification using a photometer-quantified dye feeding assay. Wild-type larvae fed either on dye alone (left) or sucrose + dye (right) for 15, 30, or 60 min. By normalizing to the dye-only group a Relative Consumption of Sucrose Index (R.C.) was calculated. Bottom: Sucrose consumption is dependent on the satiation state of larvae. An increased sucrose consumption was observed at 15 and 30 min, whereas sucrose consumption at 60 min was similar to dye-only group. (B) Representative image showing wild-type larvae fed for 15 min, 30 or 60 min either on dye or on sucrose + dye (from left, in order). (C) Top: Odor preference and high-salt avoidance assays after ingesting sucrose for 60 min. Naïve AM preference left, naïve BA preference middle, salt avoidance right. Olfactory perception was analyzed by calculating an Olfactory Preference Index (PREF). High salt avoidance was analyzed by calculating a Gustatory Avoidance Index (GAI). Bottom: Task-relevant sensory-motor abilities were not altered after sucrose consumption. Sucrose consumption, naïve odor preference, and high salt avoidance above the level of chance was tested using Bonferroni-corrected one-sample t-tests or Wilcoxon signed-rank test (ns p≥0.05/2; * p<0.05/2; adjusted significance level α). Differences between groups in (C) were determined using unpaired t-test or Mann-Whitney test. Statistically non-significant differences between groups (p≥0.05) are indicated as ns. For more statistical details see also S1 Table and S2 Table. Data are shown as Tukey box plots; line, median; cross, mean; box, 75th-25th percentiles; whiskers, 1.5 interquartile range; small circles, outlier (n≥8). AM, n-amyl acetate; BA, benzaldehyde. (TIF) [file pgen.1009064.s001.tif]

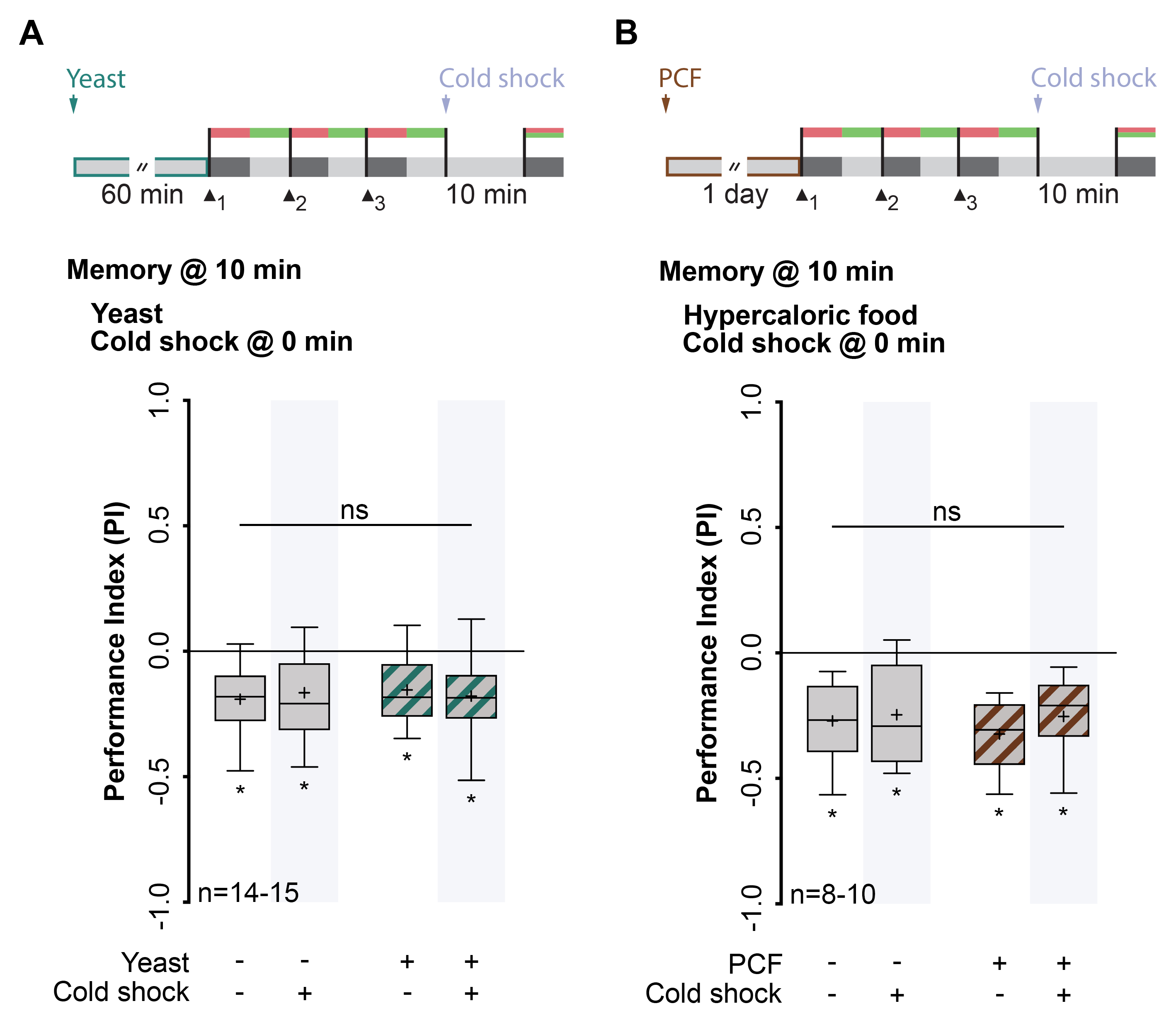

Supplement: S2 Fig — (A) Top: Training and treatment protocols. Larvae fed for 60 min on yeast and identification of lARM was carried out by applying a cold shock directly after training. Memory was tested 10 min after training. Bottom: Feeding on yeast does not inhibit lARM formation in wild-type larvae. (B) Top: Training and treatment protocols. Larvae were kept for one day on protein and carbohydrate rich food (denoted here as PCF), which is characterized by a high proportion of sucrose (150 g/l) and added pork fat (10 g/l). Identification of lARM was carried out by applying a cold shock directly after training. Memory was tested 10 minutes after training. Bottom: Feeding for one day on protein and carbohydrate rich food does not inhibit lARM formation in wild-type larvae. Memory performance above the level of chance was tested using Bonferroni-corrected one-sample t-tests (ns p≥0.05/4; * p<0.05/4; adjusted significance level α). Differences between groups were determined using two-way ANOVA followed by Bonferroni post-hoc pairwise comparisons. Statistically non-significant differences between groups (p≥0.05) are indicated as ns. For more statistical details see also S1 Table and S3 Table. Data are shown as Tukey box plots; line, median; cross, mean; box, 75th-25th percentiles; whiskers, 1.5 interquartile range; small circles, outlier (n≥8). lARM, larval anesthesia resistant memory. (TIF) [file pgen.1009064.s002.tif]

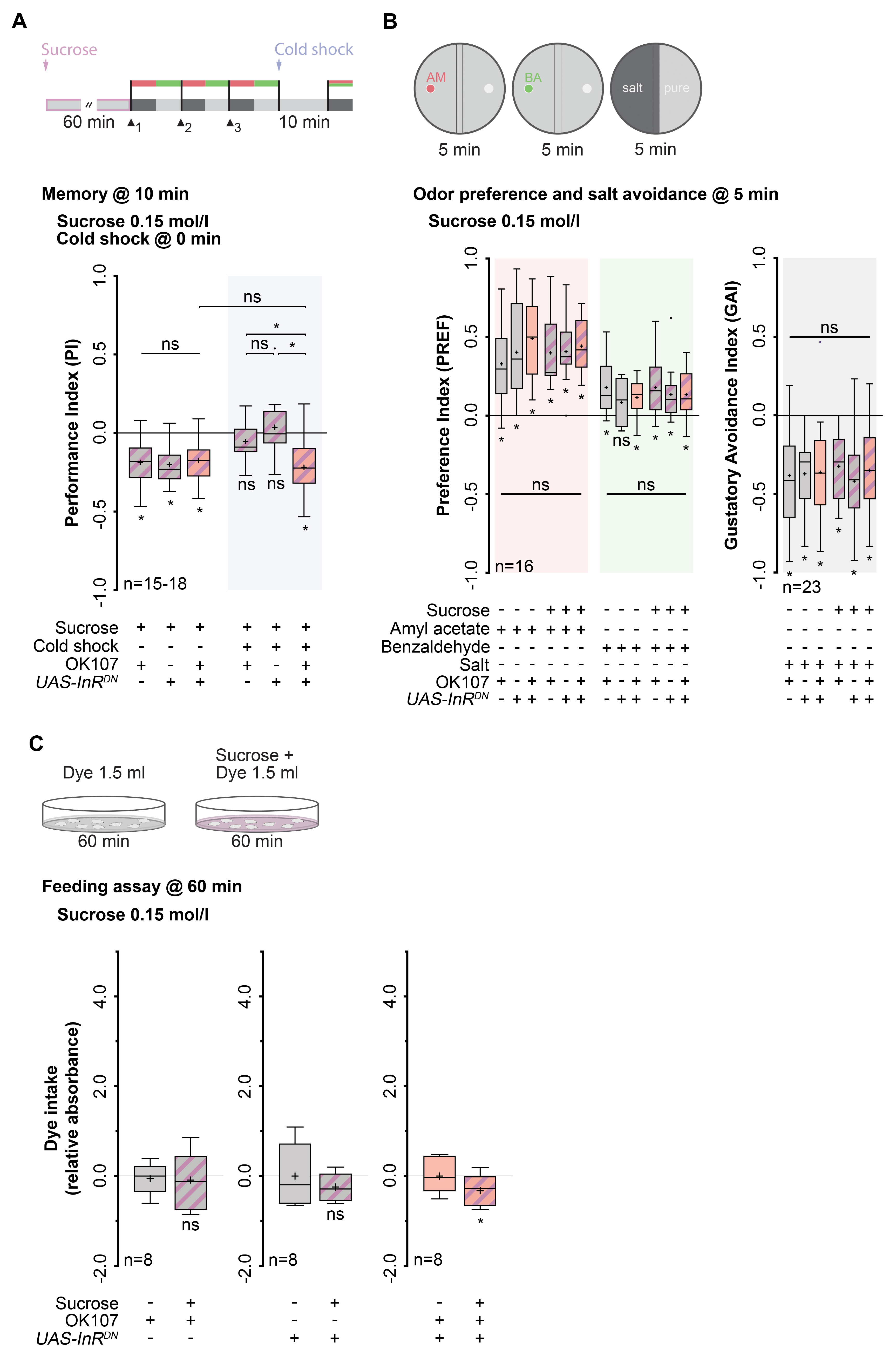

Supplement: S3 Fig — (A) Top: Training and treatment protocols. All groups consumed sucrose for 60 min and identification of lARM was carried out by applying a cold shock directly after training. Memory was tested 10 min after training. Bottom: Expression of the dominant negative form of the insulin receptor (UAS-InRDN) in KCs using the driver line OK107 prevents the suppression of lARM formation triggered by sucrose consumption. (B) Top: Odor preference and high-salt avoidance assays after ingesting sucrose for 60 min. Naïve amyl acetate (AM) left preference left, naïve benzaldehyde (BA) preference middle, salt avoidance right. Olfactory perception was analyzed by calculating an Olfactory Preference Index (PREF). High salt avoidance was analyzed by calculating a Gustatory Avoidance Index (GAI). Bottom: Task-relevant sensory-motor abilities are not altered in larvae expressing a dominant negative form of the insulin receptor (InRDN) in the MB KCs via the OK107 driver line. Bottom left: naïve odor preference for AM. Bottom middle: naïve odor preference for BA. Bottom right: naïve avoidance of a high salt concentration. (C) Top: Sucrose consumption quantification using a photometer-quantified dye feeding assay. A Relative Consumption of Sucrose Index (R.C.) was calculated by normalizing to the dye-only control. Bottom: Expression of a dominant negative insulin receptor (InRDN) transgene in MB KCs via OK107 driver line leads to a small reduction in sucrose consumption. Bottom left: Sucrose consumption in OK107/+ control group. Bottom middle: Sucrose consumption in UAS-InRDN/+ control group. Bottom right: Sucrose consumption in OK107/UAS-InRDN experimental group. For (A) and (B) memory performance, naïve odor preference and high salt avoidance above the level of chance was tested using Bonferroni-corrected one-sample t-test (ns p≥0.05/6; * p<0.05/6; adjusted significance level α). Differences between the groups were determined using two-way ANOVA followed by Bonferroni post-hoc pairwise com [file pgen.1009064.s003.tif]

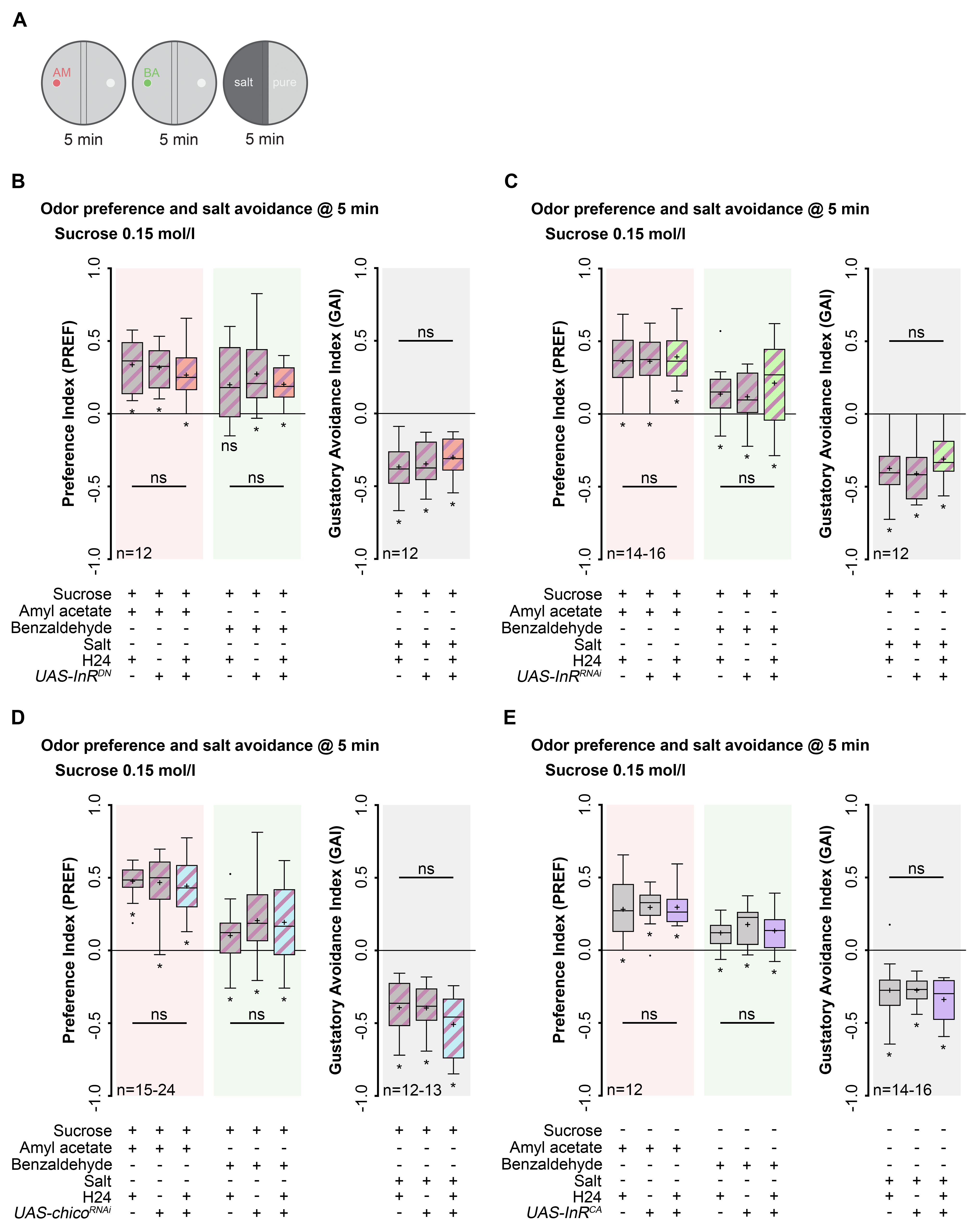

Supplement: S4 Fig — (A) Odor preference and high-salt avoidance assays after ingesting sucrose for 60 min. Naïve amyl acetate (AM) left preference left, naïve benzaldehyde (BA) preference middle, salt avoidance right. Olfactory perception was analyzed by calculating an Olfactory Preference Index (PREF). High salt avoidance was analyzed by calculating a Gustatory Avoidance Index (GAI). (B) Task-relevant sensory-motor abilities are not altered in larvae expressing a dominant negative form of the insulin receptor (InRDN) in the MB KCs via the H24 driver line. Left: naïve odor preference for AM. Middle: naïve odor preference for BA. Right: naïve avoidance of a high salt concentration. (C) Task-relevant sensory-motor abilities are not altered in larvae when knocking down insulin receptors in the MB KCs via UAS-InRRNAi and H24. Left: naïve odor preference for AM. Middle: naïve odor preference for BA. Right: naïve avoidance of a high salt concentration. (D) Task-relevant sensory-motor abilities are not altered in larvae when knocking down the Drosophila insulin receptor substrate homologue chico in the MB KCs via UAS-chicoRNAi and H24. Left: naïve odor preference for AM. Middle: naïve odor preference for BA. Right: naïve avoidance of a high salt concentration. (E) Task-relevant sensory-motor abilities are not altered in larvae expressing a constitutively active form of the InR in the MB KCs via UAS-InRCA and H24. Left: naïve odor preference for AM. Middle: naïve odor preference for BA. Right: naïve avoidance of a high salt concentration. Naïve odor preference and high salt avoidance above the level of chance was tested using Bonferroni-corrected one-sample t-test (ns p≥0.05/3; * p<0.05/3; adjusted significance level α). Differences between the groups were determined using one-way ANOVA followed by Tukey post-hoc pairwise comparisons and are depicted above the respective box plots; ns indicates p≥0.05). Data are shown as Tukey box plots; line, median; cross, mean; box, 75th-25th percentiles; w [file pgen.1009064.s004.tif]

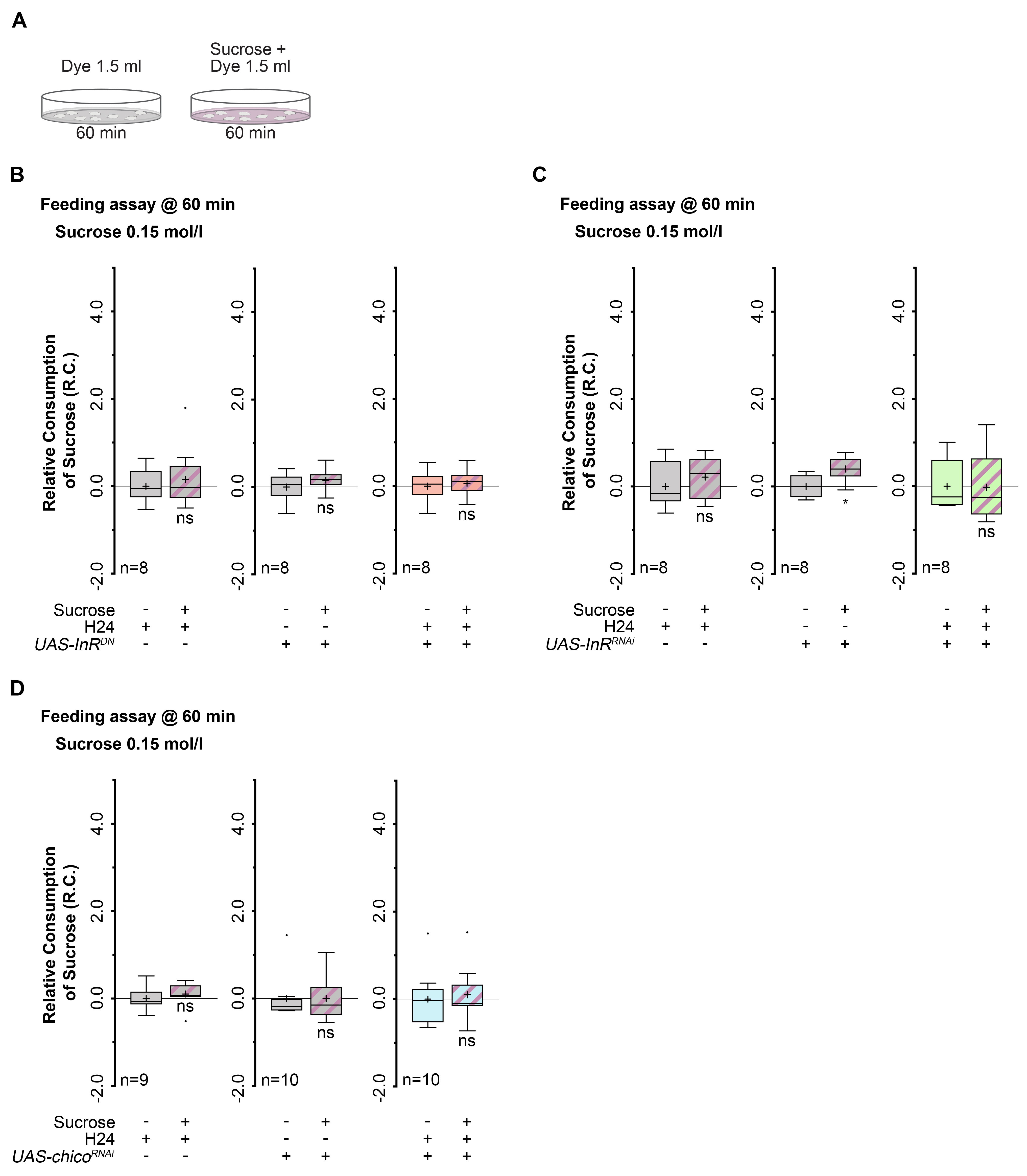

Supplement: S5 Fig — (A) Sucrose consumption quantification using a photometer-quantified dye feeding assay. A Relative Consumption of Sucrose Index (R.C.) was calculated by normalizing to the dye-only control. (B) Expression of a dominant negative insulin receptor (InRDN) transgene in MB KCs via H24 driver line does not change the sucrose consumption at 60 min. Left: Sucrose consumption in OK107/+ control group. Middle: Sucrose consumption in UAS-InRDN/+ control group. Right: Sucrose consumption in OK107/UAS-InRDN experimental group. (C) Specific knockdown of insulin receptors in MB KCs via UAS-InRRNAi and H24 does not change sucrose consumption at 60 min. Left: Sucrose consumption in OK107/+ control group. Middle: Sucrose consumption in UAS-InRRNAi/+ control group. Right: Sucrose consumption in OK107/UAS-InRRNAi experimental group. (D) Specific knockdown of the Drosophila insulin receptor substrate homologue chico in the MB KCs via UAS-chicoRNAi and H24 does not change sucrose consumption at 60 min. Left: Sucrose consumption in OK107/+ control group. Middle: Sucrose consumption in UAS-chicoRNAi/+ control group. Right: Sucrose consumption in OK107/UAS-chicoRNAi experimental group. (E) Expressing of a constitutively active form of the InR in the MB KCs via UAS-InRCA and H24 does not change sucrose consumption at 60 min. Left: Sucrose consumption in OK107/+ control group. Middle: Sucrose consumption in UAS-InRCA/+ control group. Right: Sucrose consumption in OK107/UAS-InRCA experimental group. Sucrose consumption above the level of chance was tested using Bonferroni-corrected one-sample t-tests (ns p≥0.05/2; * p<0.05/2; adjusted significance level α). For more statistical details see also S1 Table. Data are shown as Tukey box plots; line, median; cross, mean; box, 75th-25th percentiles; whiskers, 1.5 interquartile range; small circles, outlier (n≥8). CA, constitutively active; DN, dominant negative; InR, insulin receptor; KC, Kenyon cell; MB, mushroom body; UAS, upstream activation seque [file pgen.1009064.s005.tif]

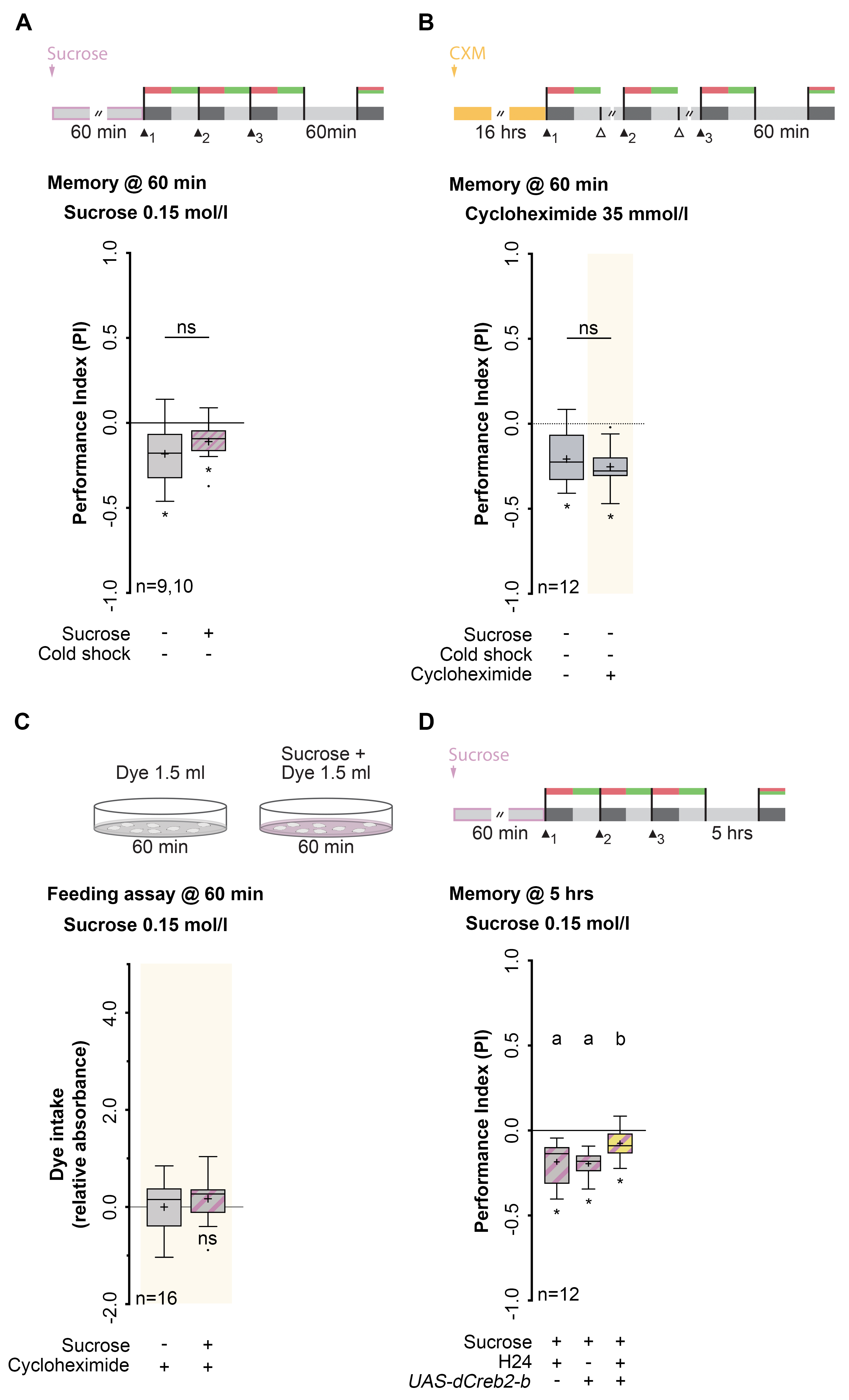

Supplement: S6 Fig — (A) Top: Training and different treatment protocols. Memory was tested 60 min after training. Bottom: Memory after sucrose consumption was statistically indistinguishable from lARM formed after being fed on tap water. (B) Top: Training and different treatment protocols. Wild-type larvae were fed CXM for 16 hours. Odor-high salt conditioning was performed using a spaced training protocol consisting of three cycles separated by 15 min rest intervals (open triangle). Memory was tested 60 min after training. Bottom: The application of CXM did not affect the aversive olfactory memory that was formed after a spaced training protocol. (C) Top: Sucrose consumption quantification in wild-type larvae using a photometer-quantified dye feeding assay. A Relative Consumption of Sucrose Index (R.C.) was calculated by normalizing to the dye-only control. Both groups were fed CXM for 16 hours before feeding on sucrose. Bottom: Sucrose consumption is not altered after CXM treatment. (D) Top: Training and treatment protocol. Memory was tested 5 hours after training, Bottom: Expression of a repressor isoform of CREB (UAS-dCREB2-b) in KCs using the driver line H24 resulted in a lower sucrose induced 5 hours memory than in both genetic control groups (H24/+ and UAS-dCREB2-b/+). For (A), (B) and (C) memory performance and sucrose consumption above the level of chance was tested using Bonferroni-corrected one-sample t-tests (ns p≥0.05/2; * p<0.05/2; adjusted significance level α). For (A) and (B) differences between groups were determined using unpaired t-test. Statistically non-significant differences between groups (p≥0.05) are indicated as ns. For (D) memory performance above the level of chance was tested using Bonferroni-corrected one-sample t-test (ns p≥0.05/3; * p<0.05/3; adjusted significance level α). Differences between the groups were determined using one-way ANOVA followed by Tukey post-hoc pairwise comparisons. Lowercase letters indicate differences between groups (p<0.05). Fo [file pgen.1009064.s006.tif]
