# Supplementary material for: Insulin signaling represents a gating mechanism between different memory phases in Drosophila larvae
Source: PLoS Genet. 2020 Oct 26;16(10):e1009064. doi: 10.1371/journal.pgen.1009064 (PMC7644093; doi:10.1371/journal.pgen.1009064)
Supplement: S2 Table — (DOCX) [file pgen.1009064.s008.docx]

### S2 Table: Statistical details of unpaired t-test or Mann-Whitney test.

|  | Statistical test | Statistical description | p-value | Significant^2^ |
| --- | --- | --- | --- | --- |
| Fig 4D | Unpaired t-test^1^ | t=2.656, df=16 | 0.017 | * |
| S1C Fig | Amyl acetate |  |  |  |
|  | Unpaired t-test^1^ | t=1.365, df=30 | 0.183 | ns |
|  | Benzaldehyde |  |  |  |
|  | Mann-Whitney test^1^ | U=122.5 | 0.846 | ns |
|  | Salt |  |  |  |
|  | Unpaired t-test^1^ | t=1.617, df=30 | 0.116 | ns |
| S6A Fig | Unpaired t-test^1^ | t=1.031, df=17 | 0.317 | ns |
| S6B Fig | Unpaired t-test^1^ | t=0.801, df=22 | 0.432 | ns |

^1^Two-tailed. ^2^Significance level was set to 𝛼=0.05. ns indicates p≥0.05, * indicates p<0.05.
