# Supplementary material for: Insulin signaling represents a gating mechanism between different memory phases in Drosophila larvae
Source: PLoS Genet. 2020 Oct 26;16(10):e1009064. doi: 10.1371/journal.pgen.1009064 (PMC7644093; doi:10.1371/journal.pgen.1009064)
Supplement: S4 Table — (DOCX) [file pgen.1009064.s010.docx]

### S4 Table: Statistical details of One-way ANOVA and Kruskal-Wallis test.

|  | Statistical description | Significant^2^ | Tukey’s multiple comparison tests^3^ | Bartlett’s test^4^ |
| --- | --- | --- | --- | --- |
| S4B Fig | Amyl acetate |  |  |  |
|  | F(2,33)=0.577, p=0.567 | ns | 1 vs 2, p=0.955, ns | p=0.689, ns |
|  |  |  | 1 vs 3, p=0.557, ns |  |
|  |  |  | 2 vs 3, p=0.736, ns |  |
|  | Benzaldehyde |  |  |  |
|  | F(2,33)=0.472, p=0.682 | ns | 1 vs 2, p=0.667, ns | p=0.078, ns |
|  |  |  | 1 vs 3, p=0.999, ns |  |
|  |  |  | 2 vs 3, p=0.695, ns |  |
|  | Salt |  |  |  |
|  | F(2,33)=0.605, p=0.552 | ns | 1 vs 2, p=0.938, ns | p=0.619, ns |
|  |  |  | 1 vs 3, p=0.536, ns |  |
|  |  |  | 2 vs 3, p=0.745, ns |  |
| S4C Fig | Amyl acetate |  |  |  |
|  | F(2,38)=0.114, p=0.893 | ns | 1 vs 2, p>0.9999, ns | p=0.531, ns |
|  |  |  | 1 vs 3, p=0.909, ns |  |
|  |  |  | 2 vs 3, p=0.910, ns |  |
|  | Benzaldehyde |  |  |  |
|  | F(2,45)=0.901, p=0.413 | ns | 1 vs 2, p=0.967, ns | p=0.160, ns |
|  |  |  | 1 vs 3, p=0.569, ns |  |
|  |  |  | 2 vs 3, p=0.421, ns |  |
|  | Salt |  |  |  |
|  | F(2,39)=1.129, p=0.334 | ns | 1 vs 2, p=0.860, ns | p=0.719, ns |
|  |  |  | 1 vs 3, p=0.607, ns |  |
|  |  |  | 2 vs 3, p=0.311, ns |  |
| S4D Fig | Amyl acetate |  |  |  |
| \|  \|  \|  \| 2 vs 3, p<0.0001. * \|  \| \| --- \| --- \| --- \| --- \| --- \| | F(2,45)=0.166, p=0.848 | ns | 1 vs 2, p=0.989, ns | p=0.094, ns |
| \|  \|  \|  \| 2 vs 3, p<0.0001. * \|  \| \| --- \| --- \| --- \| --- \| --- \| |  |  | 1 vs 3, p=0.845, ns |  |
| \|  \|  \|  \| 2 vs 3, p<0.0001. * \|  \| \| --- \| --- \| --- \| --- \| --- \| |  |  | 2 vs 3, p=0.910, ns |  |
| \|  \|  \|  \| 2 vs 3, p<0.0001. * \|  \| \| --- \| --- \| --- \| --- \| --- \| | Benzaldehyde |  |  |  |
| \|  \|  \|  \| 2 vs 3, p<0.0001. * \|  \| \| --- \| --- \| --- \| --- \| --- \| | F(2,69)=0.114, p=0.893 | ns | 1 vs 2, p=0.912, ns | p=0.727, ns |
| \|  \|  \|  \| 2 vs 3, p<0.0001. * \|  \| \| --- \| --- \| --- \| --- \| --- \| |  |  | 1 vs 3, p=0.909, ns |  |
| \|  \|  \|  \| 2 vs 3, p<0.0001. * \|  \| \| --- \| --- \| --- \| --- \| --- \| |  |  | 2 vs 3, p>0.9999, ns |  |
| \|  \|  \|  \| 2 vs 3, p<0.0001. * \|  \| \| --- \| --- \| --- \| --- \| --- \| | Salt |  |  |  |
| \|  \|  \|  \| 2 vs 3, p<0.0001. * \|  \| \| --- \| --- \| --- \| --- \| --- \| | F(2,34)1.633, p=0.210 | ns | 1 vs 2, p=0.9997, ns | p=0.635, ns |
| \|  \|  \|  \| 2 vs 3, p<0.0001. * \|  \| \| --- \| --- \| --- \| --- \| --- \| |  |  | 1 vs 3, p=0.270, ns |  |
| \|  \|  \|  \| 2 vs 3, p<0.0001. * \|  \| \| --- \| --- \| --- \| --- \| --- \| |  |  | 2 vs 3, p=0.284, ns |  |
| S4E Fig | Amyl acetate |  |  |  |
| \|  \|  \|  \| 2 vs 3, p<0.0001. * \|  \| \| --- \| --- \| --- \| --- \| --- \| | F(2,33)=0.031, p=0.969 | ns | 1 vs 2, p=0.974, ns  1 vs 3, p=0.976, ns  2 vs 3, p>0.9999, ns | p=0.201, ns |
| \|  \|  \|  \| 2 vs 3, p<0.0001. * \|  \| \| --- \| --- \| --- \| --- \| --- \| | Benzaldehyde |  |  |  |
| \|  \|  \|  \| 2 vs 3, p<0.0001. * \|  \| \| --- \| --- \| --- \| --- \| --- \| | F(2,33)=0.660, p=0.524 | ns | 1 vs 2, p=0.519, ns  1 vs 3, p=0.960, ns  2 vs 3, p=0.688, ns | p=0.553, ns |
| \|  \|  \|  \| 2 vs 3, p<0.0001. * \|  \| \| --- \| --- \| --- \| --- \| --- \| | Salt |  |  |  |
| \|  \|  \|  \| 2 vs 3, p<0.0001. * \|  \| \| --- \| --- \| --- \| --- \| --- \| | H=0.886, p=0.642 | ns | 1 vs 2, p>0.9999, ns  1 vs 3, p>0.9999, ns  2 vs 3, p>0.9999, ns | p=0.033, ns |
| S6D Fig | F(2,33)=5.704, p=0.008 | * | 1 vs 2, p=0.961, ns | p=0.158, ns |
| \|  \|  \|  \| 2 vs 3, p<0.0001. * \|  \| \| --- \| --- \| --- \| --- \| --- \| |  |  | 1 vs 3, p=0.024, *  2 vs 3, p=0.012, * |  |

^1^F(DFn, Dfd), p-value for one-way ANOVA and H, p-value for Kruskal-Wallis test. ^2^Significance level was set to 𝛼=0.05. ns indicates p≥0.05, * indicates p<0.05. ^3^Numbers correspond to boxplots from left to right. Adjusted p-values after *post-hoc* pairwise comparison. Significance level was set to 𝛼=0.05, ns indicates p≥0.05, * indicates p<0.05. ^4^Bartlett’s test for homogeneity of variances. Significance level was set to 𝛼=0.05. ns indicates p≥0.05, * indicates p<0.05
